# Witchcraft, Envy, and Norm Enforcement in Mauritius

**DOI:** 10.1007/s12110-024-09484-4

**Published:** 2025-01-22

**Authors:** Aiyana K. Willard, Nachita Rosun, Kirsten Lesage, Jan Horský, Dimitris Xygalatas

**Affiliations:** 1 Centre for Culture and Evolution, Brunel University of London, Kingston Ln, Uxbridge, UB8 3PH UK; 2 Department of Psychology, University of California, Riverside , Riverside, CA USA; 3 LEVYNA Laboratory for the Experimental Research of Religion, Masaryk University, Brno, Czechia; 4 Department of Anthropology, University of Connecticut, Mansfield, CT USA

**Keywords:** Witchcraft, Religion, Envy, Norm enforcement, Supernatural punishment, Supernatural retribution

## Abstract

Recent research has shown that an array of religious beliefs can be used to enforce socially normative behaviour, but the application of these theories to other supernatural beliefs, including witchcraft, is still nascent. Across two pre-registered studies in Mauritius, we examine how witchcraft is believed to be caused by envy and how this belief can create and enforce social norms around not causing envy. Data was collected in-person in Mauritius. In study 1 (*N* = 445), we found that both practicing witchcraft and being motivated by envy or self-interest increase perceptions of harm. These motivations also increase the rate with which people suggest a person was doing witchcraft, with envy having the stronger effect. Belief that someone was doing witchcraft increases the negativity with which one views that person and damages their reputation. In study 2 (*N* = 292), we found that when a person breaks a norm around causing envy, participants believe that a subsequent misfortune is cause by witchcraft, but not by God. When someone acts selfishly towards others a subsequent misfortune is believed to be caused by God but not witchcraft. This suggests that witchcraft beliefs, but not religious ones, are enforcing norms around preventing envy. Together, these studies suggest that witchcraft beliefs can support locally specific social norms, and that these norms might be different than those supported by religion.

**Supplementary Information:**

The online version contains supplementary material available at 10.1007/s12110-024-09484-4.

Billions of people alive today believe in witchcraft, sorcery, and other similar types of magic (Gershman, 2021). Though there is a lot of variety within these beliefs—ranging from a substance or organ in the body that causes bad things to happen to others (Evans-Pritchard, 1937) to gaining the ability to do magic through a pact with the devil (Thomas, 1973)—they have been remarkably stable across history and are common across a wide variety of cultures (Douglas, 1970; Hutton, 2017; Singh, 2021). They are still prevalent in many parts of the world today (Ashforth, 2002; Stoop & Verpoorten, 2020), including in the contemporary West (Hutton, 1999; Luhrmann, 1991; White, 2015). These beliefs have important consequences for believers, such as influencing how they think about the cause of fortune and misfortune (Willard et al., 2024), the consequence of their own and other’s actions, and how they interact with other people in their communities (Ashforth, 2005; Mace et al., 2018; Van Bastelaer & Leathers, 2006). Despite the prevalence of these magical beliefs, their relevance and impact have largely been overlooked in the cognitive and cultural evolutionary literature (but see Singh, 2021). Though some research within developmental economics has looked at their impact on trust (Gershman, 2016; Rossignol et al., 2022), little has been done to explore the psychology of these beliefs (but see Legare & Gelman, 2008).

Like many other supernatural beliefs, witchcraft beliefs have the potential to function as a form of supernatural deterrent (e.g., Kundtová Klocová et al., 2022). Whereas the more commonly studied belief in moralizing gods (gods who know all, care about what humans do, and will punish moral violations) tends to enforce broader social norms around cooperation and fairness, witchcraft beliefs are likely to enforce more specific and locally relevant social norms (e.g., Purzycki et al., 2021). Here, we are interested in how these beliefs might enforce specific norms around being showy, bragging, or otherwise doing things that might make others envious. We focus on this because of the commonly described relationship in the anthropological literature between witchcraft beliefs and envy (Douglas, 1970; Koning, 2013; Stoop & Verpoorten, 2020; Vidas, 2012). If, as we suggest below, envy is commonly believed to motivate witchcraft practitioners to curse others, then we would expect actions that cause envy, such as bragging, to be socially sanctioned because they can cause an increase in witchcraft within a community.

We define witchcraft quite broadly. We consider witchcraft to be practices and rituals in which individuals try to use magic as a supernatural force to impact others and the world.^1^ This fits with how other highly developed societies frame their own contemporary practice of witchcraft, such as in wicca or witchcraft practices in the West (Luhrmann, 1991; White, 2015), and has been the popular perception of witchcraft in Europe since the Middle Ages (Hutton, 1999). These magical abilities may be seen as positive (helping people, such as healing or protection), negative (harming people, such as curses), or a mixture of both. When witchcraft beliefs include the possibility that people can curse others and cause magical harm, they may increase distrust of others within the community (Gershman, 2021), but also could lead to a normative taboo against any behaviour that might evoke that harm—such as envy and behaviours that cause envy. These beliefs are likely also to reflect, shape, and enforce the social norms within the societies that hold them.

We are interested in the possibility that witchcraft beliefs may function to create and enforce relevant social norms, as well as in the perception and consequences of witchcraft beliefs more broadly. To these aims, we evaluate the following questions:

1. How do people perceive witchcraft and those practicing it, including how they might be causing harm?
2. Does envy as a stated motivation for witchcraft increase the perception that these practices are harmful?
3. Does behaving in a way that might evoke envy in others (i.e., bragging) increase the belief that an otherwise unrelated harm could be caused by witchcraft?

Of particular interest to us is the belief that witchcraft is motivated by envy and how this belief can shape and enforce normative behaviour around bragging, showing off, or even the willingness to tell others about one’s good fortune.

## Witchcraft, Envy, and Trust

Across the existing literature on witchcraft, envy is one of the frequently cited motivations for witchcraft (Douglas, 1970; Koning, 2013; Stoop & Verpoorten, 2020; Vidas, 2012). The most famous example may be Evans-Pritchard’s (1937) work with the Azande, where he outlines how envy, greed, and hatred are believed to be the cause of witchcraft in this community. This witchcraft is more akin to the evil eye (discussed below) than to how we are using the term here. Among the Azande, it is not a deliberate act, but rather a direct consequence of someone with the power of witchcraft experiencing these negative feelings. This distinction was not historically true of witchcraft in western contexts, where it was seen as deliberate (Sanders, 1995; Thomas, 1973), and deliberate witchcraft is found widely in other parts of Africa as well (Peacey et al., 2024). According to Evans-Pritchard (1935), these beliefs served to create and enforce norms around expressing these emotions lest one be accused of witchcraft. We are interested in the potential extension of this: that these beliefs create norms against behaving in a way that might cause others to feel envy.

Envy is seen to underly other similar beliefs, such as the evil eye (Dundes, 1981; Lykiardopoulos, 1981). Within these beliefs, when certain people envy other’s successes—such as financial success, or the birth of a healthy newborn baby—this envy causes harm to the envied individual through magical or supernatural means. In evil eye beliefs, this can be done passively. Someone’s expressed envy causes harm through bad intent without any additional actions from the envious person (Xygalatas, 2021). In our field site, it was explained to us that a stain on a new dress at a religious gathering was ultimately caused by the compliments another guest made about the dress (evil eye). This suggests that compliments are often considered disingenuous, and these beliefs can create a norm where complimenting others is seen as potentially bad behaviour. Practices like making babies ugly by putting black Kajal under their eyes and on their face in India also originate in these beliefs (Spiro, 2005; Sujatha & Rashma, 2014). In other beliefs like witchcraft, this harm can be more active where a person performs a ritual or some other act to curse another. Here, we can expect that the act of cursing itself will be sanctioned, but also potentially an act aimed at making others envious will be seen as bad behaviour because it can lead to others doing witchcraft.

Witchcraft has previously been said to play a role in social control, such as in Kluckhohn’s (1944) description of Navajo witchcraft, where leaders used accusations strategically to support their goals. This followed Malinowski (1922) in suggesting a functional role for magic within societies. Some of this literature has looked at how witchcraft beliefs may promote norms of wealth redistribution, because not giving to those less fortunate may result in envy and, therefore, a curse (Platteau, 2014; Thomas, 1970). This suggests that these beliefs play a role in the enforcement of social norms, and specifically norms around preventing envy. Further, the fear that others may cause magical harm may cause distrust, and other potentially maladaptive behaviours, such as hiding one’s own wealth and good fortune for fear of others’ reprisals or even forgoing opportunities for fear of negative outcomes (Ashforth, 2005; Gershman, 2021; Golooba-Mutebi, 2005; Green, 2005). When people believe witchcraft is the mechanism for envy-based retribution, the tendency to adopt these potentially detrimental behaviours may be amplified.

Beliefs like witchcraft are difficult to dissuade because of how they are related to common negative outcomes. If a wide array of misfortunes or illnesses are believed to be caused by witchcraft, then the existence of witchcraft is almost always confirmed (e.g., Stewart & Strathern, 2004)—all one needs is something bad to happen at some point after the possible norm violation, such as bragging in a place where this is non-normative, has occurred. These beliefs assume that some of the misfortunes and illnesses one experiences are caused by other people in the community, and thus may increase distrust of others within a community (Gershman, 2014).

## Supernatural Punishment, Supernatural Retribution, and the Impact on Social Norms

Across a wide literature, supernatural beliefs have been shown to enforce moral or social norms. When people believe that a god cares about a behaviour and will punish the behaviour, they are less likely to engage in the behaviour for fear of supernatural punishment (Norenzayan et al., 2016). Dependant on the content and context of these beliefs (Bendixen et al., 2021), supernatural punishment can promote and enforce normative behaviour ranging from broad cooperation with strangers (Lang et al., 2019; Purzycki et al., 2016) to specific norms around the division of a specific type of food within a society (Singh et al., 2020), and that different supernatural beliefs can enforce different types of norms even within a single society (Willard et al., 2020).

These acts have been largely related to prosocial or moral norms—gods care about things that help communities live and function together like generosity and fairness (Norenzayan, 2013). Witchcraft and other magic beliefs have largely been ignored in this literature, which has almost exclusively looked at non-human supernatural agents. Yet, these magical beliefs may also impact the creation and enforcement of social norms. In the case of witchcraft, we are interested to see if these norm enforcement properties extend to a different type of supernatural belief, those where the supernatural act is more retributive than moral.

Social norms are, by definition, behavioural rules that others in your community will punish you for breaking, and that punishment will be socially sanctioned (Chudek & Henrich, 2011). When this punishment is outsourced to a deity, we call it supernatural punishment. Retributive acts are those aimed to inflict harm on others because of a perceived slight or upset. Curses and magical harm are a type of supernatural retribution, where a practitioner causes harm to someone they see as deserving of admonishment. Previous research in Mauritius has found that people are more likely to suggest that an illness was potentially cause by witchcraft if they know the sufferer behaved badly towards another in the past (Willard et al., 2024).

Destructive envy is well known to lead retributive or destructive acts (Smith & Kim, 2007). In this sense, envy-motivated witchcraft is a type of supernatural retribution (Gershman, 2014). Similar to supernatural punishment, supernatural retribution may support and enforce normative behaviours if people believe that retribution is caused by the victim is breaking a social norm. Here also, a behaviour may be become normatively sanctioned because it can cause others to engage in witchcraft. For example, bragging may become a norm violation because it can lead to one getting cursed, in a similar way to how the threat of divine punishment can make an act like praying normative.

## Witchcraft in Mauritius

Mauritius is a small, highly developed, high-income, and multicultural island nation in the Indian Ocean. The main industries are manufacturing, tourism, and finance. Much of this development has happened in the past 50 years since its independence, meaning that different generations currently alive in Mauritius have grown up in vastly different socioeconomic conditions and have differing average levels of education. Mauritius has no indigenous population. The current population are largely the descendants of enslaved people, indentured labourers, or other people brought to the island by the French or British during colonial rule. The existing population consists of Indian-origin Hindus and Muslims, African-origin people, and smaller groups of Chinese-, and European-origin people. The dominant religious group are Hindus, but the approach of the government and the people of Mauritius is one of religious pluralism and tolerance. Mauritians routinely participate in festivals and celebrations of other religious groups and live in a mixed society with low conflict among groups.

With this, there is some mixing of practices and traditions at the edges of society. Witchcraft beliefs in Mauritius combine Hindu, traditional African, and Christian icons and beliefs (Čaval, 2018; Chazan-Gillig & Ramhota, 2023). These practices involve complex ritual practices, and practitioners seek training, with training in Madagascar and other off-island places seen as more prestigious (see Kundtová Klocová et al., 2022). There are a variety of magic professionals in Mauritius. ‘*Traiteurs*’ specialise in healing. ‘*Traiteur*’ is a broad term applied to all types of faith healers and those that can remove a curse, evil eye, or black magic. *Ruqya* practitioners specialise in exorcisms and the removal of *jinn* in an Islamic context. ‘*Longanistes*’ are black magic practitioners (though sometimes this label of ‘black magic’ is also applied to *traiteurs*). Despite these categories, any magic professional could be involved in both healing and black magic practices and the distinction is often not clear cut.

The practices our team most frequently came across consisted of trapping the souls of the dead to do the practitioner’s bidding. This is practiced by both Hindu and Creole practitioners, though our informants on this topic were predominantly Hindu. We are unsure about its prevalence in the Muslim population as we received mixed claims. *Ruqya* is commonly practiced. The soul is believed to exit the body upon death and live on as a spirit (*nam*). Sorcerers try to capture those souls by various means, such as offering prayers and sacrifices to the Grand Croix (Big Cross) in a graveyard to gain control of them. Once under the influence of a sorcerer, the soul becomes a *jab* (evil spirit), and can be summoned to do the bidding of the practitioner through special rituals and offerings such as rum and cigarettes (a practice found elsewhere in Africa), as well as fruit sacrifices similar to those found in Hinduism. Distressed souls are the best for these purposes, so souls of past slaves or those who have died in a distressing way are most sought after.

Though these practices are taboo and illegal in Mauritius, there is widespread knowledge of their existence. Most Mauritians are aware of common locations where these practices take place (graveyards, abandoned religious spaces, and crossroads) as well as individuals who practice them, and can easily recognise the remnants of magic practices routinely found in these places. Knowledge about the use of crossroads as a site for witchcraft practices is especially pervasive as the phrase ‘*lor lacroisée*’ (on the crossroad) is commonly used to indicate witchcraft. Services from *traiteurs* and *longanistes* are frequently advertised through posters in public spaces, such as bus stops, and on social media. These services are widely available to paying customers, though it is difficult to get an estimate of how much they are used. Neither practitioners nor clients actively disclose this information, particularly when it is related to a curse or vendetta. The practitioners that we did speak to all claimed that they never curse others, only help or remove curses, but also that they make good money at their practice. It is clear, based on the remnants of rituals found in graveyards and other places (e.g., rusty nails and other items wrapped in black or red cloth; lemons with nails through them), that curses are being practiced by many. Though the gender distribution of magical practitioners is not known to us, all the practitioners we talked to were male (see Peacey et al., 2022).

Local newspapers have recently written about these services as a form of scam (Defi Media, 2023). Recently, some practitioners have been exposed on Facebook and other social media platforms and people are sometimes arrested (e.g., Vencatareddy-Nursingen, 2024). Beyond legal consequences, gossip about who engages in witchcraft is widespread in Mauritius, which can lead to reputational damage and loss of social support. Still, compared to other parts of Africa, such as Gambia and Ghana, accusations in Mauritius are relatively infrequent and mild. The accusations we were most often exposed to were somewhat casual claims that neighbours had potentially cursed others in the neighbourhood, but in at least one instance led to an eviction. This types of gossip and accusation is also sometimes weaponised as a type of character assassination in other sorts of disputes (see Stewart & Strathern, 2004), such as legal disputes.

## Research Objectives

The objective of this project is to gain a better understanding of the impact of witchcraft beliefs in Mauritius, with a focus on how they might increase perceptions of distrust and harm, and if they play a role in enforcing norms around envy and not making others envious. We accomplish this by using a mixed methodology that combines two vignette studies and a free-list task assessing how participants think about witches and witchcraft. Our vignettes present people with scenarios in which we manipulate a character’s actions and their motivations for doing those actions. These manipulations allowed us to assess whether either action or motivation increase the perception of witchcraft and change the perceived consequences of an act. Mauritius was chosen for this research because of the commonness of the belief in, and practice of witchcraft, and because, unlike many places where these beliefs are common, the taboo around these beliefs is not strong enough to prevent people from talking with us about them.

The specific objectives vary between the two studies, but together they aim to illustrate that people believe envy can motivate harmful witchcraft practices, and these beliefs can police actions that could make others envious because of the perceived harm caused by witchcraft. We are further interested in whether these beliefs impact interpersonal trust in these communities.

## Study 1

The aim of this study was to test (1) how people perceive witchcraft and those practicing it, including how they might be causing harm and (2) whether envy as a stated motivation increases the perception that these practices are harmful rather than helpful. To do this, we assessed the conditions under which witchcraft and envy might be perceived to cause harm and distrust, and to see how these relationships interacted. We used vignettes paired with pictures to suggest a motivation (neutral, self-interest, envy) for doing a religious ritual, a magic ritual, or no ritual. Self-interest, as an additional motivation, allows us to compare envy against another common motivation for engaging in these activities. We included religion to compare witchcraft to another type of common supernatural belief that is known to enforce normative behaviour in Mauritius (Xygalatas et al., 2013, 2016). This allowed us to assess the influence of both the action and the motivation on the perceptions of people conducting these acts.

Our pre-registered predictions were that participants who were given vignettes about (a) magic and (b) envy would see the action as more harmful to the community and rate the perpetrators of these acts as less trustworthy and generally more negatively. We also predicted that vignette stories where the character was doing a religious ritual would offer some protection against the negative impacts of envy as a motivation, thus participants in the religious condition would see envy as having lower negative consequences than in the other conditions. Envy as a motivation to go to the temple and pray should not have the same connotations as envy as a direct motivation for witchcraft and may be seem as a more constructive way of dealing with envy.

We also tested some exploratory hypotheses, specifically that participants would be more likely to think the action presented in the vignette was witchcraft when we suggested the character’s motivation was envy. We look at the perceived effectiveness of these practices by asking about the impact on the practitioner and the potential target. We suggested two possible directions of perceived harm of these vignette actions. If participants’ answers are based entirely on the belief that these rituals work, then they should rate the magic and envy conditions as (a) helping the character performing the ritual and harming the supposed victim. On the other hand, if ratings are driven by an understanding of the taboo around magic, then participants should rate (b) as more harmful to the character performing the ritual rather than the supposed victim.

## Methods

Data for this study were collected in 2018 and pre-registered. Pre-registrations, materials and data are available at: https://osf.io/rsb6f/. Ethical approval was granted through Brunel University of London.

## Participants

Participants (*N* = 445, 51.5% female)^2^ were recruited on streets, bus stops, and beaches across the island of Mauritius and interviewed by research assistants. The average age was 40.39 (range 18–79, *SD* = 16.20). Participants had an average of 12.1 years of formal education (range: 0–26 years; *SD* = 3.95). Education was negatively correlated with age, with older participants having a lower average education than younger ones (*r* = − 0.40, 95% CI [− 0.48 to − 0.32]). All participants were Hindu (including Tamil, Telegu, Marathi, and mainstream Hindus). We sampled only Hindus to keep the rituals and photos consistent in the religious ritual condition, and because Hindus are the largest demographic group in Mauritius.

## Materials and Procedures

Participants were asked some basic demographic questions before being read a short vignette and shown a photo. We used a factorial design, with 3 vignettes and 3 photos combined into 9 conditions (Table 1). Each participant only saw one of the nine conditions. All interviews were conducted in Mauritian Creole by local research assistants, and questionnaire data were entered on tablets while the interviews took place. Free-lists were collected on paper, and data were entered after the interviews by the interviewer. Each interview took approximately 7–10 min.

**Table 1:** Vignette conditions 3 × 3 design

	Secular	Religious	Magic
Neutral	1. No motivation + Secular setting/picture	2. No motivation + Religious setting/picture	3. No motivation + Magic setting/picture
Self interest	4. Self-interest + Secular setting/picture	5. Self-interest + Religious setting/picture	6. Self-interest + Magic setting/picture
Envy	7. Envy + Secular setting/picture	8. Envy + Religious setting/picture	9. Envy + Magic setting/picture

### Vignettes

The three vignettes covered three emotional states used to convey 3 possible motivations. In all vignettes, participants were introduced to a man who owns a business that he recently moved to a new location. Motivations were (1) *a neutral condition*, in which this is all the information that was given; (2) *a self-interest condition*, in which participants were told that the man’s business was not doing well and he wanted his business to be more successful; and (3) *an envy condition*, in which participants were told that another business was doing better than this man’s business, he was envious, and wanted his business to be more successful. Each of these conditions was paired with one of three pictures with some additional information.

### Pictures

**Fig. 1:**
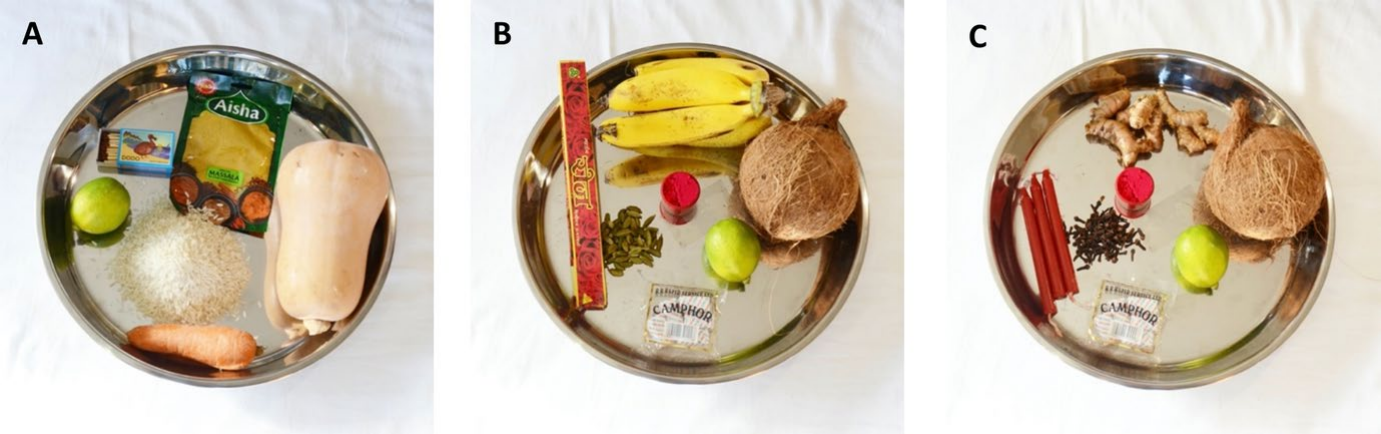
Pictures used to illustrate ritual action. (**A**) Secular/neutral condition, (**B**) Religious condition, (**C**) Witchcraft/magic condition

The picture conditions cover three sets of actions (Fig. 1): (A) The secular/neutral condition included a photo depicting a tray of food and participants were told that the man took this food home for his wife to cook; (B) The religious condition included a photo depicting a tray of food and items typically used in a religious ritual (*puja*) and participants were told that the man bought these to take to the temple; (C) The witchcraft/Magic condition included a photo depicting items typically used in magic rituals, and participants were told the man purchased these to bring them to a crossroad. There was no mention of rituals in the vignettes, and the photos in the experimental conditions would have looked indistinguishable to an unfamiliar eye, but Mauritians would have readily recognized their different uses in temple rituals and witchcraft, respectively.

### Scaled Questions

After the vignettes, participants were asked three Likert scale questions about the consequences of the man’s actions for (1) his own business, (2) other businesses, and (3) the community (e.g., how good or bad were the man’s actions for his own business? ). These questions were on a 5-point scale, where the midpoint was given last (i.e., extremely bad, bad, good, extremely good, or neither good nor bad).^3^ This was done to reduce a tendency in Mauritius to favour the midpoint of the scale for these types of questions. The inclusion of own business and other business gives us some insight into beliefs about how effective these practices are. If they are seen as widely effective, they should benefit the practitioner’s own business and harm the other business. These were followed by 6 questions assessing the qualities of the man himself (Were his actions helpful? Were his actions harmful? Was he trustworthy? Did the participant like him? Did he seem like a good person? Did he seem like a selfish person? ). These questions were asked on a 4-point scale with no midpoint to encourage people to make positive or negative judgments.

### Free-list and Open-Ended Questions

Participants were asked to fill in three free-list questions, where they were asked to list up to three possible answers to each question:

1. What are the possible reasons the man did the action shown in the photo?
2. What are the possible outcomes of the actions the man performed in the photo?
3. What do you think of the man?

Finally, participants were asked an open-ended question about what they thought they saw in the photo.

### Demographics

In addition to age, gender, and education, four questions were included to assess socioeconomic status (i.e., profession, houses owned, cars owned, and fluency in English), and religiosity. Income was not included as it is considered impolite to ask about income directly in Mauritius. The frequency of ritual practices was also requested but not included in any of the models because of its overlap with religious belief.

## Results

Data from the Likert scale questions were analysed using Bayesian linear regressions with weakly normalizing priors (see ESM). Interaction effects between all the picture and motivation conditions were included to test whether motivation effects differed across each picture condition. Age (divided by 10 so coefficients are easier to interpret) and gender were included in all models. Models with additional demographic controls for years of education, religiosity, and SES can be found in the ESM. The inclusion of these demographic variables does not change the interpretation of any of the results presented below.

### Are Witchcraft or Religious Practices and their Motivations Believed to Help or Hinder?

To assess whether participants saw these practices and motivations as helping or hindering people and the community, we looked at how the conditions impacted answers to questions about consequences to one’s own business, other businesses, and the community (Table 2). Negative estimates show higher perceived harm, and more positive estimates show higher perceived help. The neutral motivation in the secular condition (intercepts) is rated as somewhat positive (helping) across all questions. The magic condition is rated more negatively (harming) for all questions, though this effect is notably larger for the man’s own business and for the community, suggesting that participants claim these practices harm the practitioner more than the potential target. Similarly, the religion condition leads to a positive effect for the man’s own business and community, with a smaller positive effect for his own business and a credibility interval that contains 0, suggesting that participants claim these practices are generally helpful. Similar patterns are seen for the motivations, with self-interested motivations leading to negative effects across all conditions, but envy only leading to sizable negative effects for the man’s own business and for the community.

**Table 2:** Condition predicting ratings of helping/harming the character’s own business, the other character’s business, or the community (5-point scale). Model 1 is without interactions between ritual and motivation. Model 2 includes these interactions

	Own business		Other business		Community	
	Model 1	Model 2	Model 1	Model 2	Model 1	Model 2
Predictors	Estimates (95% CI)	Estimates (95% CI)	Estimates (95% CI)	Estimates (95% CI)	Estimates (95% CI)	Estimates (95% CI)
Intercept	0.55*	0.66*	0.33*	0.37*	0.43*	0.47*
	(0.33 to 0.76)	(0.40 to 0.91)	(0.14 to 0.53)	(0.13 to 0.63)	(0.24 to 0.62)	(0.23 to 0.70)
Magic	−0.98*	−1.25*	−0.58*	−0.73*	−1.13*	−1.29*
	(− 1.20 to − 0.77)	(− 1.60 to − 0.88)	(− 0.79 to − 0.36)	(− 1.09 to − 0.37)	(− 1.32 to − 0.93)	(− 1.62 to − 0.96)
Religion	0.51*	0.43*	0.19†	0.21	0.33*	0.36*
	(0.29 to 0.72)	(0.08 to 0.79)	(− 0.02 to 0.40)	(− 0.14 to 0.56)	(0.13 to 0.53)	(0.04 to 0.69)
Envy	−0.55*	−0.73*	−0.26*	−0.22	−0.47*	−0.41*
	(− 0.76 to − 0.32)	(− 1.08 to − 0.39)	(− 0.47 to − 0.04)	(− 0.56 to 0.11)	(− 0.67 to − 0.28)	(− 0.72 to − 0.07)
Self interest	−0.38*	−0.53*	−0.30*	−0.44*	−0.27*	−0.45*
	(− 0.60 to − 0.17)	(− 0.88 to − 0.19)	(− 0.51 to − 0.08)	(− 0.78 to − 0.11)	(− 0.46 to − 0.07)	(− 0.78 to − 0.13)
Age	0.08*	0.08*	0.01	0.01	0.04	0.04
	(0.03 to 0.13)	(0.03 to 0.14)	(− 0.04 to 0.07)	(− 0.04 to 0.07)	(− 0.02 to 0.09)	(− 0.01 to 0.09)
Male	0.03	0.04	0.06	0.06	0.13	0.13
	(− 0.15 to 0.20)	(− 0.15 to 0.21)	(− 0.12 to 0.24)	(− 0.11 to 0.23)	(− 0.04 to 0.30)	(− 0.04 to 0.30)
Magic*Envy		0.51*		0.15		0.21
		(0.01 to 1.00)		(− 0.34 to 0.65)		(− 0.27 to 0.64)
Religion*Envy		0.06		−0.24		−0.38
		(− 0.44 to 0.56)		(− 0.71 to 0.25)		(− 0.85 to 0.06)
Magic*Selfish		0.28		0.30		0.30
		(− 0.24 to 0.79)		(− 0.19 to 0.79)		(− 0.16 to 0.76)
Religion*Selfish		0.19		0.17		0.29
		(− 0.32 to 0.67)		(− 0.31 to 0.67)		(− 0.16 to 0.75)
Observations	429		429		429	

*95% credible interval does not cross 0

**Fig. 2:**
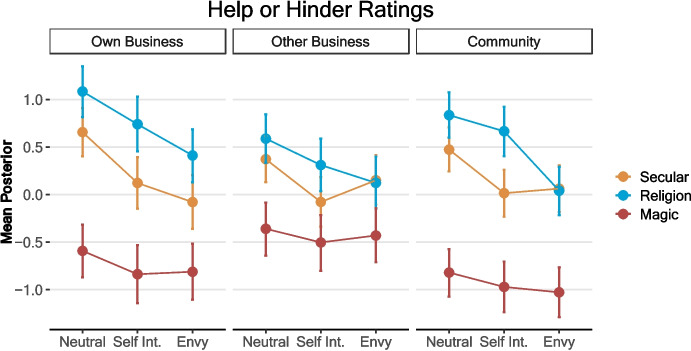
Helping and hindering ratings. Error bars are 95% credibility intervals of the posterior

Across all questions, interaction effects were small, with the largest effect being for envy in the magic condition. Envy was related to a lower increase in negative ratings here than in the secular condition, likely because the magic condition was already seen as quite negative (Fig. 2). We see no evidence of a protective effect of religion on envy, but rather envy is seen as relatively more negative in this condition.

### Are the People Practicing Witchcraft or Religion Seen as more or less Trustworthy? As Positive or Negative?

We looked at ratings of the vignette character in two ways. As it was one of our main variables of interest, we looked at trust separately from the other items. Since they were highly intercorrelated (α = 0.86, 95% CI [0.84 to 0.88]), all other items were looked at together in a multi-level model (Table 3). Harmful and selfish questions were reversed so that higher ratings always meant a more positive perception of the vignette character. Trust was highly correlated with many of these items (correlation coefficients between 0.53 and 0.75), and it is likely that the effects of all come from the same underlying positive or negative evaluation of the person (see ESM for each effect analysed individually).

**Table 3:** Conditions predicting rating of trusting the character, and a combined score for all other person ratings (4-point Likert scale). Model 1 is without interactions between ritual and motivation. Model 2 includes these interactions

	Trust		Other person ratings	
	Model 1	Model 2	Model 1	Model 2
Predictors	Estimates (95% CI)	Estimates (95% CI)	Estimates (95% CI)	Estimates (95% CI)
Intercept	−0.08	−0.01	0.23	0.25
	(− 0.23 to 0.07)	(− 0.20 to 0.19)	(− 0.08 to 0.60)	(− 0.04 to 0.58)
Magic	−0.66*	−0.75*	−0.72*	−0.77*
	(− 0.82 to − 0.50)	(− 1.02 to − 0.48)	(− 0.79 to − 0.64)	(− 0.90 to − 0.64)
Religion	0.14	0.03	0.09*	0.07
	(− 0.02 to 0.30)	(− 0.24 to 0.29)	(0.01 to 0.16)	(− 0.06 to 0.20)
Envy	−0.22*	−0.30*	−0.30*	−0.33*
	(− 0.37 to − 0.07)	(− 0.57 to − 0.04)	(− 0.38 to − 0.23)	(− 0.45 to − 0.20)
Selfish Interest	−0.08	−0.21	−0.13*	−0.18*
	(− 0.23 to 0.08)	(− 0.47 to 0.06)	(− 0.21 to − 0.06)	(− 0.31 to − 0.05)
Age	0.06*	0.06*	0.04*	0.04*
	(0.02 to 0.10)	(0.02 to 0.10)	(0.02 to 0.05)	(0.02 to 0.06)
Male	0.07	0.07	0.10*	0.10*
	(− 0.07 to 0.20)	(− 0.07 to 0.20)	(0.04 to 0.16)	(0.04 to 0.16)
Magic*Envy		0.16		0.10
		(− 0.22 to 0.53)		(− 0.08 to 0.27)
Religion*Envy		0.08		−0.05
		(− 0.28 to 0.44)		(− 0.22 to 0.14)
Magic*Self Int.		0.12		0.06
		(− 0.25 to 0.50)		(− 0.12 to 0.23)
Religion*Self Int.		0.27		0.10
		(− 0.12 to 0.64)		(− 0.08 to 0.28)
Observations	413	413	2010	2010
Random Effects				
σ^2^			0.46	0.46
τ_00_			0.1	0.1
ICC			0.18	0.18
N			5	5

*95% credible interval does not cross 0

**Fig. 3:**
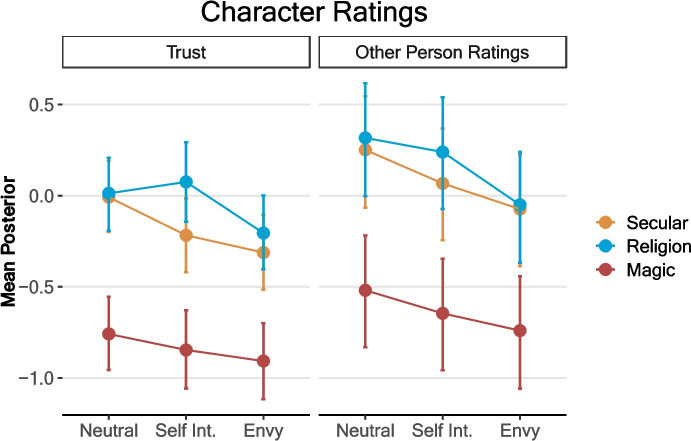
Character ratings. Error bars are 95% credible intervals of the posterior

Both the magic and envy conditions led to lower trust and more negative overall evaluations of the character (Fig. 3). Self-interested motivations also had a negative impact on trust and other evaluations, but these effects were somewhat smaller. The religion condition had no overall impact. The impact of motivation did not differ substantially in the magic or religion condition (though the religion condition was notably higher than the secular condition for self-interest in the trust ratings). Again, we see no indication of a protective effect of religion on the perceptions of envy.

## Exploratory Analysis

Free-lists were coded by two independent coders with good reliability (Cohen’s Kappa = 0.75 [95% CI: 0.56 to 0.94] for free-lists; 100% agreement on witchcraft accusations, and 97.5% agreement of suggestions of witchcraft). Coders were blind to the conditions. When coders disagreed, the final codes were decided by the first author. Free-lists were analysed for salience using Smith’s S calculated using the AnthroTools package in R (Purzycki & Jamieson-Lane, 2017). This is done by weighing items based on where they appear in the list (the first item is given a higher weight than the second, the second is weighted higher than the third, etc.). Individual item salience is calculated with the following equation:

$$\:i=\frac{n+1-k}{n}$$

Here, *n* is the total number of items in a participant’s list, and *k* is the order in which that item was listed. Smith’s S is calculated as the mean salience value of all items (*i*) of that type (Smith, 1993):

$$\:S=\:\frac{\sum\:{i}_{T}}{N}$$

This allows us to give more weight to the first thing people think of than the second or third. Salience scores above 0.10 for each question are shown in the figures below.

### Accusations of Witchcraft

Across the three free-list questions and the final open-ended question, we coded for claims and suggestions that the vignette character was doing witchcraft. Participants were coded as claiming witchcraft if they said that the man was doing black magic, witchcraft, sorcery, or he was a *longaniste.* Participants were further said to be suggesting witchcraft if they claimed he was doing evil, bad things, or other suggestions that what he was doing had evil connotations. We found that even in the neutral (secular) and religion conditions, a substantial portion of people claimed or suggested that the character was doing witchcraft and that this increased when the character was given a motivation (Table 4).

**Table 4:** Percentage who claimed or suggested that the story character was doing witchcraft

		Secular	Religion	Magic
Claimed witchcraft	Neutral	5.4%	5.4%	47.2%
	Self interest	15.0%	0.0%	38.9%
	Envy	17.1%	7.8%	50.0%
Claimed or suggested witchcraft	Neutral	8.1%	10.1%	77.8%
	Self interest	30.0%	8.1%	88.9%
	Envy	36.6%	30.8%	89.5%

We analysed these data to see whether the picture conditions or motivations predicted when people suggested or claimed the man was doing witchcraft (Table 5). Accusations of witchcraft were, unsurprisingly, predicted by the magic condition but not the religious condition. Envy was also a strong predictor of witchcraft accusations. Self-interest showed a sizable but smaller increase in the secular condition. Though none of the interaction effects were reliably predicted here, only envy increased accusations of witchcraft in all conditions, and self-interest did not increase accusations in the religion condition.

**Table 5:** Odds of suggesting the character was doing witchcraft by condition. Model 1 is without interactions between ritual and motivation. Model 2 includes these interactions

	Model 1		Model 2	
Predictors	OR	CI (95%)	OR	CI (95%)
Intercept	0.21*	0.11 to 0.39	0.22*	0.11 to 0.40
Magic	16.65*	8.91 to 32.98	14.19*	6.32 to 32.02
Religion	0.53*	0.29 to 0.99	0.55	0.22 to 1.32
Envy	3.17*	1.67 to 6.06	2.66*	1.27 to 5.76
Self interest	1.86†	0.96 to 3.50	1.88	0.85 to 4.08
Age	0.89	0.75 to 1.05	0.88	0.75 to 1.04
Male	0.81	0.47 to 1.40	0.83	0.49 to 1.43
Magic*Envy			1.15	0.36 to 3.78
Religion*Envy			1.41	0.46 to 4.23
Magic*Self int.			1.59	0.52 to 5.29
Religion*Self int.			0.49	0.14 to 1.60
Observations	341		341	

*95% credible interval does not cross 1

### Free-list Analyses

#### Why did the Man do what was Shown in the Photo?

**Fig. 4:**
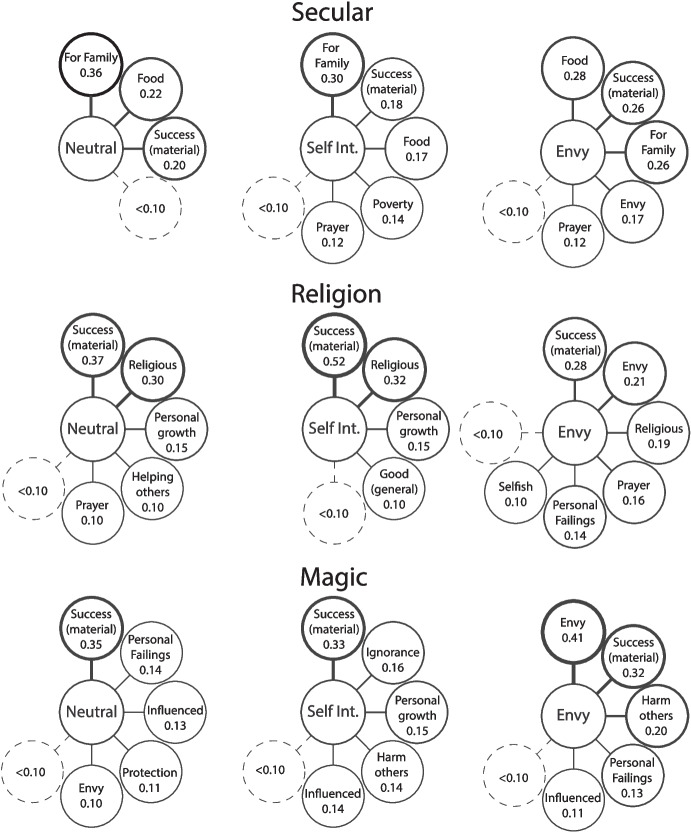
Free-list salience scores for the question “Why did the man do what was shown in the photo?” Line thickness indicates salience

For the first question about why the man did the actions shown in the photo, material success was one of the most prominent motivations across all conditions (Fig. 4). Importantly, this was consistent in both the religion and magic conditions. The other motivations seen in the magic condition tend towards the more negative. Ignorance, personal failings (such as being weak-willed or cowardly), and a desire to harm others all made an appearance. Being influenced by others (with negative connotations) was also prominent. Envy was listed as a motivation in all the envy conditions. Personal failings were also listed in the envy-religion condition despite not appearing with high salience in any other religion lists. Like the witchcraft accusations analysis above, this suggests that envy is associated with negative or bad motivations even when not explicitly associated with witchcraft.

#### What are some Consequences of his Actions?

**Fig. 5:**
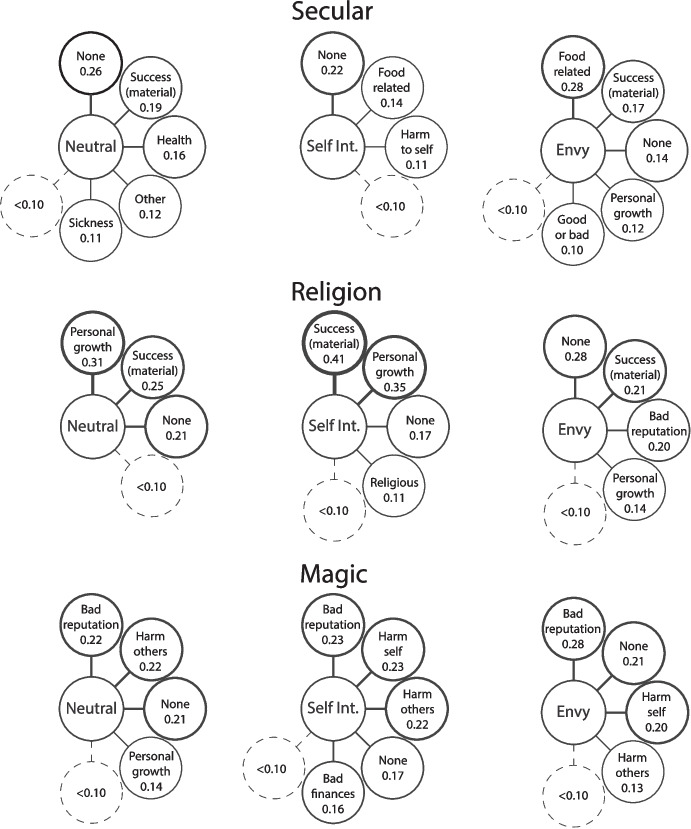
Free-list salience scores for the question “What are some consequences of his actions?” Line thickness indicates salience

For the second question asking about the consequences of the man’s actions, the most obvious result is the salience of reputational damage in the magic condition (Fig. 5). This is the highest salience item in all three motivation conditions. Harm to self and others also appears across the lists, suggesting that, overall, people think that these actions will have bad outcomes. Personal growth does appear in the neutral magic list, which may be indicative of the commonness of these practices and some amount of belief that these practices are not always bad. Bad reputation appears in the religion-envy list as well. This supports the previous analysis in evidencing that envy as a motivation increased the likelihood that the vignette character is potentially doing something bad in his ritual.

None or no consequences appear in all lists. This is less surprising in the secular condition but may suggest some level of expressed scepticism of the efficacy of these actions in the religion and magic conditions.

#### What is your Opinion of the Man?

**Fig. 6:**
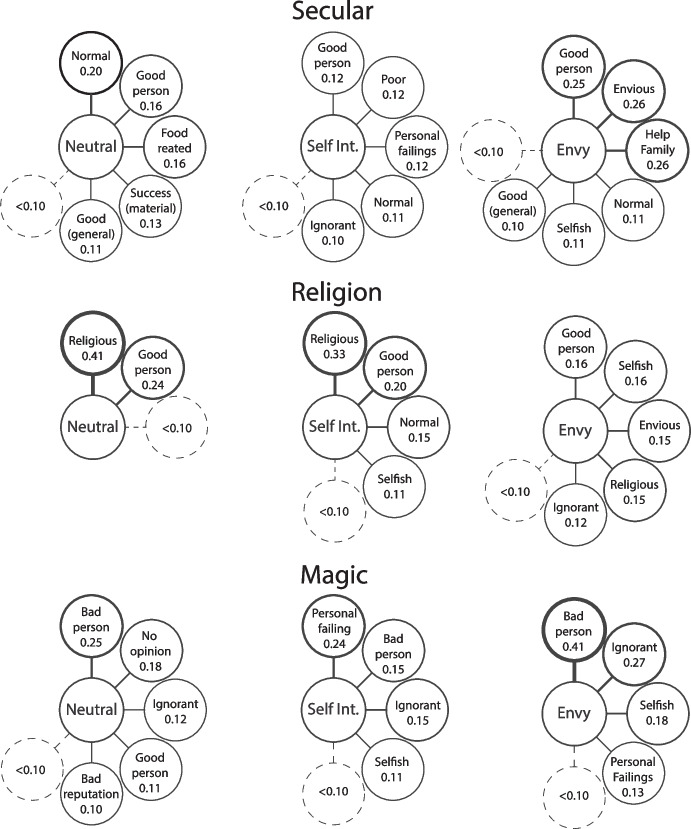
Free-list salience scores for the question “What is your opinion of the man?” Line thickness indicates salience

The final free-list question follows a similar pattern to those above, with the magic conditions including largely negative responses and some negative responses becoming more salient in the non-magic envy conditions (Fig. 6). All magic conditions had ‘bad person’ and ‘ignorant’ as salient items. ‘Ignorant’ also appeared in the religion-envy list, along with ‘selfish’ and ‘envious’. It is worth noting that all items in the religion-envy list have a low salience, meaning that no item was particularly common across participants’ lists, suggesting a high level of disagreement. The salience of ‘bad person’ in the magic lists can be contrasted with the salience of ‘good person’ in all other lists. The clear bias in this population is to refer to a person positively when relying on limited information and talking to a stranger.

## Discussion

Across all analyses, practicing witchcraft is seen as predominantly negative, and motivation matters in determining how negatively one’s actions are viewed. We found this to be true both for perceptions of harm and for the negativity of rating of the practitioners themselves. Motivations had less of an effect in the magic condition than in the secular (neutral) and religion conditions. This is likely due to the overall negative ratings in the magic condition. The free-list answers support this perspective, where the opinions of the story character expressed in all the magic conditions are predominantly negative. Though we do find the predicted effects for trust, where magic and envy would cause participants to rate the character as less trustworthy, these results may similarly reflect an overall increase in the negative view of the character, rather than be about trust specifically.

The main claimed consequence of practicing magic in the free-list data was a bad reputation. This, along with harm to oneself, indicates that people are more comfortable suggesting that the practitioner themselves will be harmed more than the potential victims of a curse. The frequency of ‘none’ as a free-list answer also suggests a claimed lack of belief in the efficacy of these acts. The claim of a bad reputation has negative consequences for cooperation and can increase a sense of distrust. Seeing this magic practitioner as a bad person will likely reduce others’ willingness to interact, share, trade, or otherwise help the character.

We find no evidence that the religion condition reduced the negative associations with envy, though this lack of effect may be because a large number of people who saw the religious-envy condition decided that the vignette character was not in fact going to a temple to pray, but rather going somewhere else to do witchcraft. The finding that envy as a motivation increased the claim that a person is doing witchcraft, even in the non-magic conditions, was not anticipated. This suggests that knowing someone is envious will create suspicion of witchcraft among others and should create a strong motivation to avoid being seen as an envious person. We expand on this in our second study by looking at how causing envy may increase the likelihood that participants would see harm as being caused by witchcraft. These findings fit with the existing anthropological literature in suggesting that envy is seen as a primary motivation for witchcraft, and when someone is known to be envious, they are more likely to be accused as a witch. As far as we are aware, this is the first time these predictions have been quantitatively tested.

## Study 2

In our second study, we build on the findings from the first study—that witchcraft is believed to be caused by envy and that envy increased the perceived harms of witchcraft—by examining whether these beliefs can enforce specific envy-related norms in a society. Specifically, we were interested in whether and how norms aimed at preventing envy in others, such as norms against bragging or being showy when one has good fortune, are enforced through fear of retribution through witchcraft. The belief that engaging in these behaviours could get a person cursed should encourage people to follow these norms and increase the belief that breaking these norms will have a negative effect. As in the previous study, we look at the impact of these beliefs on interpersonal trust. Rather than look at trust in any specific individual, we are interested in whether the belief that other people in your community can cause magical harm will negatively impact people’s general trust in their community members.

Our aim in this study was to test whether the belief that harm was caused by witchcraft increased when a vignette character broke a norm around envy (e.g., being showy or bragging). We looked at the belief in supernatural harm in two ways: first, by directly asking questions about God and witchcraft as potential causes, and second, by indirectly asking participants whether other people who behaved in the same way would come to similar harm. Since witchcraft is still a somewhat taboo topic in Mauritius, this second option allows for people who are less willing to claim witchcraft as a cause to still express a perception that breaking the norm can cause otherwise unrelated harm.

## Methods

Data for this study was collected in late 2022 and preregistered. Pre-registrations, materials and data are available at: https://osf.io/5c3dy/. Ethical approval was granted through Brunel University of London.

## Participants

Participants were recruited on the streets of several towns in Mauritius and interviewed by research assistants (*N* = 292, 50.96% female). Unlike the previous study, these data were collected across all religious groups (128 Hindus, 56 Muslims, 95 Christians, 11 others, and 5 prefer not to say). The average age was 39.18 (range 18–78, SD = 15.96). We did not collect education data.

## Materials and Procedures

All interviews were conducted in Mauritian Creole, and data were entered by research assistants on tablet computers while the interviews were taking place. Participants were read 3 vignettes and asked 6 Likert-type questions about each of them. Each interview took approximately 5 min.

### Vignettes

We created 3 sets of vignettes, each with 3 different normative/non-normative conditions (9 vignettes in total, but only 3 conditions). This was done to allow for a within-subject design where each participant saw all three conditions. Three different stories were created with 3 different characters (Rahul, Sandra, and Ali; see ESM) who all experience misfortune. The conditions presented were derived from relevant norms in the local context. The vignette conditions were as follows:

1. *Envy condition*: Bragging by being overly showy with new wealth (Sandra and Rahul) or bragging about getting into a prestigious overseas school which would lead to more wealth in the future (Ali).
2. *Selfish condition*: Getting new wealth/getting into school and not helping or intending to not help people in your family who need money.
3. *Normative condition*: The vignette character acquired new wealth and followed the appropriate local norms (did not tell those outside their immediate family about it and spent money/reported success cautiously in a non-showy way).

For the misfortunes:

1. *Sandra* got a chronic illness.
2. *Rahul* had a car accident and wrecked his car.
3. *Ali* broke his legs.

### Vignette Questions

Each vignette was followed by 6 questions. To assess whether the action broke a norm, we asked (1) whether what the character did was good or bad. To look at the cause in a more indirect way, we asked (2) whether the misfortune was the vignette character’s fault and (3) whether someone else doing the same thing would come to similar harm. For a more direct look at the cause, we asked how likely it was that the misfortune was caused by (4) God and (5) someone trying to harm the character through magic. Finally, we asked how much participants (6) would trust people in the character’s community.

These questions allow us to assess whether (a) the action itself is seen as bad, (b) there is an expectation of supernatural punishment for the action and from where, and (c) there are broader implications for trust in the community when people think that magic might be the cause.

### Demographics

Minimal demographics were asked in this survey: we collected data on age, gender, ethnicity, religion, and religiosity. Religion, but not ethnicity, was used in the models. This is because there is a large amount of overlap in these groups, and religion is a more reliable cue of group differences in beliefs.

## Results

Results were analysed using multilevel Bayesian linear regression models with weakly normalizing priors. Random slopes were included for participants to account for the non-independence of data points.^4^ Each question was analysed separately with condition, age, gender, and religiosity included. Age (divided by 10) and gender were included in all models. Models with additional controls for religiosity and religious group can be found in the ESM.

We looked at how good or bad participants rated the character’s behaviour to make sure that we were capturing social norm violations. Participants saw the normative behaviour as positive on average (Est. = 1.48; 95% CI:1.14 to 1.85), and both selfishness (Est. = −0.97; 95% CI: −1.29 to − 0.64) and inducing envy (Est. = −3.27; 95% CI: −3.59 to − 2.94) were seen as more negative (full table in ESM). It is worth noting here that this leaves the average rating for the selfish conditions as slightly positive (*simple slope* = 0.52; 95% CI: 0.19 to 0.88), whereas the envy condition was, on average, fairly negative (*simple slope* = − 1.77; 95% CI: −2.10 to − 1.44). These effects were consistent in pattern and direction when the vignettes are looked at individually but are weaker for both the selfishness and envy conditions in Ali’s vignette (who got into an overseas school) than the other two vignettes (see ESM).

### Indirect Indications of Supernatural Punishment

**Fig. 7:**
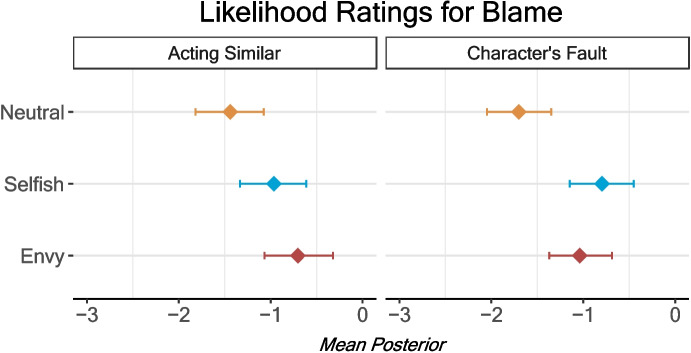
Condition predicting blaming one’s self and belief that the someone else doing similar behaviour would experience the same issues. Error bars are 95% credible interval of the posterior

To examine more indirect questions of cause, we asked participants whether they blamed the victim and whether they thought someone else acting similarly would also come to harm. We found that both the envy and selfish conditions increased how likely participants were to claim the character’s misfortune was their own fault (Table 6). In contrast to the ratings for how good or bad the behaviour was outlined above, the effect here was larger for the selfish condition than the envy condition. Participants similarly rated both conditions as increasing the likelihood that another person behaving in the same way would have a similar outcome (Fig. 7). This suggests that people connect the characters’ misfortune to their norm-violating actions, even when a direct cause is made implausible (i.e., bragging about your car cannot cause a car crash by any natural means).

**Table 6:** Condition predicting blaming one’s self and belief that the someone else doing similar behaviour would experience the same issues (7-point Likert scale)

	Own fault	Someone acting similar
Predictors	Estimates (95% CI)	Estimates (95% CI)
Intercept	−1.70*	−1.43*
	(− 2.05 to − 1.35)	(− 1.79 to − 1.07)
Envy	0.67*	0.73*
	(0.35 to 0.97)	(0.46 to 1.02)
Selfish	0.91*	0.47*
	(0.60 to 1.22)	(0.18 to 0.76)
Age	0.06	0.08
	(− 0.04 to 0.17)	(− 0.04 to 0.21)
Male	−0.03	−0.04
	(− 0.35 to 0.31)	(− 0.43 to 0.35)
Ali	−0.46*	0.07
	(− 0.77 to − 0.15)	(− 0.23 to 0.35)
Sandra	−0.25†	0.00
	(− 0.57 to 0.05)	(− 0.28 to 0.28)
Observations	722	726

*95% credible interval does not cross 0

### God and Magic as Causes

**Fig. 8:**
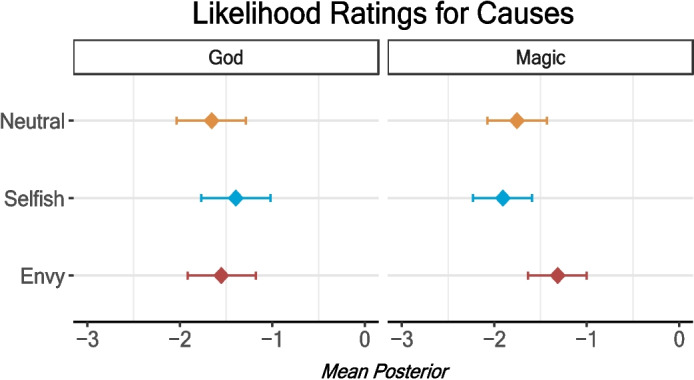
Condition predicting cause ratings for God and Magic (7-point Likert scale). Error bars are 95% credible intervals of the posterior

Ratings of how likely the misfortune was caused by God were increased by the selfish condition but not substantially by the envy condition (Table 7). We see the opposite and more extreme version of this pattern when we look at ratings of how likely the misfortune was caused by witchcraft (magic). Here, the increase was only in the envy condition and not at all in the selfish condition (Fig. 8). This suggests witchcraft is believed to punish social norm violations but that it is used for different norms than belief in God.

**Table 7:** Condition predicting ratings for God and Magic as a cause (7-point Likert scale)

	God	Magic
Predictors	Estimates (95% CI)	Estimates (95% CI)
Intercept	−1.65*	−1.76*
	(− 2.02 to − 1.27)	(− 2.07 to − 1.44)
Envy	0.10	0.44*
	(− 0.14 to 0.33)	(0.24 to 0.64)
Selfish	0.26*	−0.15
	(0.02 to 0.49)	(− 0.36 to 0.05)
Age	−0.12†	−0.10†
	(− 0.27 to 0.02)	(− 0.22 to 0.01)
Male	0.11	−0.19
	(− 0.34 to 0.56)	(− 0.56 to 0.20)
Ali	0.26*	−0.10
	(0.03 to 0.50)	(− 0.30 to 0.10)
Sandra	0.19	−0.14
	(− 0.04 to 0.42)	(− 0.34 to 0.06)
Observations	722	726

*95% credible interval does not cross 0

### Effects on Trust

We found no effects of condition for trust in the community. Overall, people showed a general lack of trust even in the normative condition (Est. = −1.63; 95% CI: −1.96 to − 1.32), but this was not notably decreased in either the selfish (Est. = −0.10; 95% CI: −0.28 to 0.07) or the envy conditions (Est. = −0.10; 95% CI: −0.28 to 0.07). When likelihood ratings for magic are included in the model, we similarly see no reliable or sizable decreases in trust with an increased belief that the harm was caused by witchcraft (Est. = −0.05; 95% CI: −0.12 to 0.01). See ESM for full tables.

## Discussion

Here, we find evidence that when a person has violated a social norm around bragging or otherwise causing envy in others, an unrelated misfortune is more likely to be believed to be caused by witchcraft. Behaving in a way that could cause envy in others, though seen as overall quite negative, is not believed to be punished by God. In contrast, we see no evidence that witchcraft-based punishment is believed to be directed at those who are selfish, but God is seen as slightly more likely to be the cause of misfortune here. Together, this is good evidence that these two types of supernatural punishment enforce different types of normative behaviours—supernatural punishment by God can make people less likely to act selfishly (Norenzayan, 2013), and fear of supernatural retribution through witchcraft can make people less likely to brag or be showy. Further, in our sample, participants rate envy norm violations as much worse and more likely to cause the same harm to others if they violate the same norm. Similar effects are found for selfish behaviour, but these effects were smaller. This suggests that people expect these acts to receive some type of supernatural punishment or retribution.

### General Discussion

Across these two studies, we find evidence that in Mauritius envy is believed to be a motivation for witchcraft and that this belief helps enforce norms against bragging or being showy (i.e., norms against behaviours that cause envy). In study 1, this was most clearly observed in our exploratory analysis where we found that envy increased the rate at which participants were willing to claim a character was doing witchcraft, even when told he was doing something else such as a religious ritual. When given the chance to give open-ended answers, around one third of participants indicated that the character might be doing magic in both non-magic envy conditions (36.6% in the secular condition and 30% in the religion condition). Similar results were seen in the self-interest condition, but only in the secular condition (secular 30%; religion 8.1%). This may be because doing a religious ritual to improve one’s business is well within the bounds of normal religious practice, but this claim comes across as suspicious when no ritual is stated. Still, these effects are strong evidence that people see witchcraft as motivated by envy and, to a lesser degree, by self-interest and that people in this population are very willing to assume that a person is doing witchcraft on motivational evidence alone. This is supported by the findings of increased perceived harm and more negative person ratings when the character is envious or when more explicitly doing witchcraft.

In study 2, both selfishness and envy were rated more negatively than a normative action. Moreover, they predict higher rates of blaming the victim for their own misfortune and the likelihood of claiming that someone else behaving in the same manner would similarly come to harm. More relevantly, participants saw these two non-normative behaviours of envy and selfishness as supernaturally admonished in different ways. In the envy condition, magical retribution was rated as a more likely cause of the characters’ misfortune, but not God. The reverse is seen in the selfish condition, where God’s punishment was seen as a cause, but magic was not. This evidence suggests that these different supernatural beliefs are related to the enforcement, through perceived punishment or retribution, of different social norms.

These norms around the control of envy may be overlooked in the literature because of their lack of prominence in certain Western cultures, where talking about your accomplishments is often encouraged. In other parts of the world, these norms and beliefs are common, for example, Richard Lee’s (1984) work where he discusses his gift of meat being denigrated by the !Kung as a social levelling mechanism aimed at preventing pride and jealousy.

Envy-based magic beliefs may have widespread implications for the societies that hold them, particularly in areas of social capital which require interpersonal trust. Social capital, including trusting others, is an important part of well-functioning communities (Algan & Cahuc, 2010; Woolcock, 1998). Without trust, it is difficult to build the sort of reciprocal relationships required for cooperative groups (Tanis & Postmes, 2005) and functional markets (Arrow, 1972). The erosion of social capital through negatively valenced witchcraft beliefs can have consequences for the long-term development of a society (Gershman, 2016). Though we did not find good evidence here of trust varying with our manipulations, overall trust was quite low—people on average said they did not trust others—and the impact of the belief in witchcraft on trust may reflect a general lack of trust in this society, rather than a lack of trust brought on by the knowledge of a specific act of magic as presented in our vignettes. Regardless, the open-ended free-list responses suggested that a person believed to be doing witchcraft would suffer reputational damage and, more generally, be seen as a bad person. This is an indication that people would refrain from interacting and cooperating with these individuals. This type of social isolation of those labelled ‘witches’ has been seen elsewhere, where the social and trade networks of these witches are nearly completely separate from those of non-witches (Mace et al., 2018).

The role of these norms in society has been theorised as a way to prevent interpersonal issues that can come from inequalities in close-knit communities (Gershman, 2013; Stoop & Verpoorten, 2020). This may be particularly important in places where resources are limited or, more specifically, zero-sum. In these situations, having a larger proportion of finite resources means others get less. It has been previously hypothesised that witchcraft beliefs and their roots in envy and envy prevention can be a way to reduce the perceived value of accruing wealth much beyond the average wealth of those around you (Gershman, 2014). This is not the current economic situation in Mauritius, which in recent decades experienced rapid economic growth. It falls into the same developmental category as Europe, with a similar developmental index to Serbia (which also has high rates of witchcraft beliefs and practitioners; Čvorović, 2009, 2013). It is possible that these beliefs are a hold-over from a more resource-poor time, but this raises the question of why these beliefs persist when economic conditions that may have loaned them some function have declined.

Witchcraft beliefs are often portrayed in the anthropological literature as only negative (Singh, 2021), and the term itself often only refers to the negative uses of magic. Some of this slant may come from a Christian skewed perspective where these beliefs in Europe have been associated with devil worship and evil, but is not how these beliefs are portrayed in all cultures (Hutton, 2017), and it is worth noting that this skew may have lessened or disappeared if the broader definition we employ in this paper was used in this previous work. Though in many places, including Mauritius, the practice of witchcraft is seen as predominantly negative, it is also the case that people who use these practices are also believed to do positive things such as bring good fortune or heal. People who practice magic in Mauritius claim they do it to help others deal with a wide array of problems and improve their lives. Though this may still be seen as an improper means of getting help, more work needs to be done to look at the perceptions of magic when it is claimed to be done for more positive purposes. This also makes it clear that envy is not the only, or possibly not even the most common, motivation for these magic practices. Broadly speaking, the belief that other people can have magical powers, trained or innately, is largely missing from the evolutionary and cognitive science literature despite being a common theme in the ethnographic literature.

These beliefs do not seem to be declining—in fact, they may even be increasing. In the West, this can be seen with the increasing public presence of Wicca and other witchcraft practices on social media and in popular culture (White, 2015). Though Wicca may be a very different sort of practice to those practiced in Africa, Asia, and elsewhere, the same principle of humans using magic to influence other people and the world is at its core. This type of belief is a topic ripe for further research and will help us better understand the full spectrum of supernatural beliefs we see in human cultures and how they impact and are impacted by the societies we live in.

## Conclusions

This paper falls within a growing body of literature that shows the interaction between belief systems and the ecologies that hold them (Lansing, 2012; Purzycki, 2016; Singh et al., 2020; Willard et al., 2020; Xygalatas et al., 2022; Xygalatas & Maňo, 2022). Across this literature, it is becoming increasingly clear that the role of different beliefs within a culture maps onto that culture’s social and ecological context (Purzycki & Sosis, 2009). We add to this literature by finding that witchcraft beliefs in Mauritius function to support norms around not causing envy through the belief that you will be susceptible to witchcraft. Further, showing envy may make others worry that you will be causing them harm through magical means. The consequences of witchcraft beliefs in societies that hold them are inevitably much broader than this and the normative behaviour these beliefs enforce is unlikely to be limited to envy. Given the widespread presence of these beliefs across a large number of social and ecological contexts, much more work needs to be done exploring these impacts.

## Supplementary Information

Below is the link to the electronic supplementary material.

ESM 1 (DOCX 95.2 KB)

## Data Availability

Data, materials, and pre-registration for study 1 are available here: https://osf.io/rsb6f/. Data, materials, and pre-registration for study 2 are available here: https://osf.io/5c3dy/.

## Footnotes

1 Within some of the literature and in some communities, this would be considered sorcery and not witchcraft, but this is not consistent and we see no distinction between sorcery and witchcraft in Mauritius where this research was conducted.

2 We preregistered a sample of 336 but were able to collect more. The pre-registered estimate was based on what we thought was reasonable to collect in the time we had for data collection. This turned out to be an underestimate.

3 This scale midpoint was added after initial pilots as some participants requested a neutral option for these questions.

4 This deviates from out pre-registration, where we planned to include intercepts for stories rather than participants. This was changed because of convergence issues with the story intercepts. Estimates remain the same, but intervals change.
