# Supplementary material for: Witchcraft, Envy, and Norm Enforcement in Mauritius
Source: Hum Nat. 2025 Jan 22;35(4):347–81. doi: 10.1007/s12110-024-09484-4 (PMC11836218; doi:10.1007/s12110-024-09484-4)
Supplement: Supplementary file 1 — (DOCX 95.2 KB) [file 12110_2024_9484_MOESM1_ESM.docx]

Witchcraft, Envy, and Norm Enforcement in Mauritius

Aiyana K. Willard*, Nachita Rosun, Kirsten Lesage, Jan Horský, Dimitris Xygalatas

*corresponding author: Email: aiyana.willard@brunel.ac.uk

Published in *Human Nature* 35(4), 2024. https://doi.org/10.1007/s12110-024-09484-4

Table of Contents

[Study 1 2](#_Toc184204268)

[Vignettes 2](#_Toc184204269)

[Neutral - Cooking 2](#_Toc184204270)

[Neutral - Religion 2](#_Toc184204271)

[Neutral - Magic 2](#_Toc184204272)

[Envy - Cooking 2](#_Toc184204273)

[Envy - Religion 2](#_Toc184204274)

[Envy - Magic 2](#_Toc184204275)

[Self interest - Cooking 2](#_Toc184204276)

[Self interest - Religion 2](#_Toc184204277)

[Self interest - Magic 2](#_Toc184204278)

[Priors 3](#_Toc184204279)

[Questions 3](#_Toc184204280)

[Tables 4](#_Toc184204281)

[Other Business, Own Business, and Community 4](#_Toc184204282)

[Trust and Character Rating Tables 7](#_Toc184204283)

[Witchcraft accusation tables 10](#_Toc184204284)

[Study 2 12](#_Toc184204285)

[Vignettes 12](#_Toc184204286)

[Rahul – Normative 12](#_Toc184204287)

[Rahul – Envy 12](#_Toc184204288)

[Rahul – Selfish 12](#_Toc184204289)

[Sandra – Normative 12](#_Toc184204290)

[Sandra – Envy 12](#_Toc184204291)

[Sandra – Selfish 12](#_Toc184204292)

[Ali – Normative 12](#_Toc184204293)

[Ali – Envy 12](#_Toc184204294)

[Ali – Selfish 12](#_Toc184204295)

[Questions 13](#_Toc184204296)

[Priors 13](#_Toc184204297)

[Tables 13](#_Toc184204298)

# Study 1

## Vignettes

### *Neutral - Cooking*

The man who bought the following items is a business owner.

He recently moved his business to a new location in his village.

He bought these items for his wife to cook.

### Neutral - Religion

The man who bought the following items is a business owner.

He recently moved his business to a new location in his village.

He bought these items to bring to the temple.

### Neutral - Magic

The man who bought the following items is a business owner.

He recently moved his business to a new location in his village.

He bought these items to bring to the cross roads.

### Envy - Cooking

The man who commissioned bought the following items is a business owner. There is another business near him that is similar to his business. This business is more successful than his business. He is jealous of this business and would like his business to be more successful.

He recently moved his business to a new location in his village.

He bought these items for his wife to cook.

### Envy - Religion

The man who commissioned bought the following items is a business owner. There is another business near him that is similar to his business. This business is more successful than his business. He is jealous of this business and would like his business to be more successful.

He recently moved his business to a new location in his village.

He bought these items to bring to the temple.

### Envy - Magic

The man who commissioned bought the following items is a business owner. There is another business near him that is similar to his business. This business is more successful than his business. He is jealous of this business and would like his business to be more successful.

He recently moved his business to a new location in his village.

He bought these items to bring to the cross roads.

### Self interest - Cooking

The man who bought the following items is a business owner. His business has not been doing very well lately. He would like his business to be more successful.

He recently moved his business to a new location in his village.

He bought these items for his wife to cook.

### Self interest - Religion

The man who bought the following items is a business owner. His business has not been doing very well lately. He would like his business to be more successful.

He recently moved his business to a new location in his village.

He bought these items to bring to the temple.

### Self interest - Magic

The man who bought the following items is a business owner. His business has not been doing very well lately. He would like his business to be more successful.

He recently moved his business to a new location in his village.

He bought these items to bring to the cross roads.

## Priors

For most models:

$$\alpha\sim Normal\left( 0,1 \right)$$

$$\beta\sim Normal\left( 0,1 \right)$$

For multilevel models:

$$\alpha\sim Normal\left( 0,1 \right)$$

$$\beta\sim Normal\left( 0,1 \right)$$

$$\sigma^{2}\sim Exponential(1)$$

## Questions

Help/harm

1. What will be the consequences of the man's actions for other businesses? (similar to the man's business). [Other Businesses, 1-5 Extremely bad to Extremely good]
2. What will be the consequences of the man's actions for his business? [Own Businesses, 1-5 Extremely bad to Extremely good]
3. What will be the consequences of the man's actions for the man's community? [Community, 1-5 Extremely bad to Extremely good]

Character ratings

1. I would trust this man [Trust, 1-4 Strongly disagree to strongly agree]
2. The man’s actions were helpful [Helpful, 1-4 Strongly disagree to strongly agree]
3. I like this man [Like, 1-4 Strongly disagree to strongly agree]
4. The man’s actions were harmful [harmful, 1-4 Strongly disagree to strongly agree]
5. This man seems like a selfish person. [Selfish, 1-4 Strongly disagree to strongly agree]

## Tables

Tables include models 1) without interaction effects; 2) with interaction effects; and 3) with additional socio-economic variables and religiosity. For all tables, * means that the 95% Credibility Interval does not include 0 (or 1 logistic models), and † means that the 90% Credibility Interval does not include 0 (or 1 logistic models).

### Other Business, Own Business, and Community

Table 1: Regression predicting perceived help or harm to other businesses [-2 to +2 Likert scale] from vignette condition

| **Other Business** | **Model 1** | | | | **Model 2** | | | | **Model 3** | | | |
| --- | --- | --- | --- | --- | --- | --- | --- | --- | --- | --- | --- | --- |
| ***Predictors*** | ***Est.*** | ***SE*** | ***CI (95%)*** | ***CI (90%)*** | ***Est.*** | ***SE*** | ***CI (95%)*** | ***CI (90%)*** | ***Est.*** | ***SE*** | ***CI (95%)*** | ***CI (90%)*** |
| Intercept | 0.33* | 0.10 | 0.14 – 0.53 | 0.17 – 0.50 | 0.37* | 0.13 | 0.13 – 0.63 | 0.17 – 0.58 | 0.22 | 0.22 | -0.22 – 0.65 | -0.15 – 0.59 |
| Magic | -0.58* | 0.11 | -0.79 – -0.36 | -0.77 – -0.40 | -0.73* | 0.18 | -1.09 – -0.37 | -1.03 – -0.45 | -0.78* | 0.19 | -1.15 – -0.41 | -1.09 – -0.47 |
| Religion | 0.19† | 0.11 | -0.02 – 0.40 | 0.01 – 0.37 | 0.21 | 0.17 | -0.14 – 0.56 | -0.08 – 0.50 | 0.18 | 0.18 | -0.18 – 0.54 | -0.12 – 0.48 |
| Envy | -0.26* | 0.11 | -0.47 – -0.04 | -0.43 – -0.08 | -0.22 | 0.18 | -0.56 – 0.11 | -0.51 – 0.06 | -0.19 | 0.19 | -0.55 – 0.16 | -0.50 – 0.10 |
| Self Interest | -0.30* | 0.11 | -0.51 – -0.08 | -0.47 – -0.11 | -0.44* | 0.17 | -0.78 – -0.11 | -0.73 – -0.16 | -0.42* | 0.18 | -0.77 – -0.06 | -0.72 – -0.11 |
| Age | 0.01 | 0.03 | -0.04 – 0.07 | -0.04 – 0.06 | 0.01 | 0.03 | -0.04 – 0.07 | -0.03 – 0.06 | 0.01 | 0.03 | -0.05 – 0.07 | -0.05 – 0.06 |
| Male | 0.06 | 0.09 | -0.12 – 0.24 | -0.09 – 0.21 | 0.06 | 0.09 | -0.11 – 0.23 | -0.08 – 0.21 | 0.03 | 0.1 | -0.16 – 0.21 | -0.13 – 0.19 |
| Magic*Envy |  |  |  |  | 0.15 | 0.24 | -0.34 – 0.65 | -0.26 – 0.56 | 0.12 | 0.26 | -0.38 – 0.61 | -0.30 – 0.54 |
| Religion*Envy |  |  |  |  | -0.24 | 0.25 | -0.71 – 0.25 | -0.64 – 0.17 | -0.25 | 0.26 | -0.74 – 0.27 | -0.66 – 0.18 |
| Magic*Self Int |  |  |  |  | 0.30 | 0.26 | -0.19 – 0.79 | -0.10 – 0.73 | 0.31 | 0.26 | -0.20 – 0.82 | -0.11 – 0.74 |
| Religion*Self Int |  |  |  |  | 0.17 | 0.25 | -0.31 – 0.67 | -0.23 – 0.59 | 0.14 | 0.25 | -0.36 – 0.64 | -0.29 – 0.55 |
| Education |  |  |  |  |  |  |  |  | 0.01 | 0.02 | -0.03 – 0.04 | -0.02 – 0.04 |
| Religiosity |  |  |  |  |  |  |  |  | -0.09 | 0.05 | -0.19 – 0.02 | -0.18 – 0.00 |
| Houses owned |  |  |  |  |  |  |  |  | 0.00 | 0.07 | -0.14 – 0.12 | -0.12 – 0.11 |
| Cars owned |  |  |  |  |  |  |  |  | -0.05 | 0.04 | -0.13 – 0.03 | -0.12 – 0.02 |
| English |  |  |  |  |  |  |  |  | 0.16 | 0.1 | -0.04 – 0.36 | -0.01 – 0.33 |
| Observations | 429 |  |  |  | 429 |  |  |  | 401 |  |  |  |

Table 2: Regression predicting perceived help or harm to character’s own businesses [-2 to +2 Likert scale] from vignette condition

| **Own Business** | **Model 1** | | | | **Model 2** | | | | **Model 3** | | | |
| --- | --- | --- | --- | --- | --- | --- | --- | --- | --- | --- | --- | --- |
| ***Predictors*** | ***Est.*** | ***SE*** | ***CI (95%)*** | ***CI (90%)*** | ***Est.*** | ***SE*** | ***CI (95%)*** | ***CI (90%)*** | ***Est.*** | ***SE*** | ***CI (95%)*** | ***CI (90%)*** |
| Intercept | 0.55* | 0.10 | 0.33 – 0.76 | 0.37 – 0.72 | 0.66* | 0.13 | 0.40 – 0.91 | 0.44 – 0.87 | 0.10 | 0.23 | -0.35 – 0.56 | -0.27 – 0.50 |
| Magic | -0.98* | 0.11 | -1.20 – -0.77 | -1.16 – -0.80 | -1.25* | 0.18 | -1.60 – -0.88 | -1.54 – -0.94 | -1.28* | 0.18 | -1.66 – -0.92 | -1.59 – -0.97 |
| Religion | 0.51* | 0.11 | 0.29 – 0.72 | 0.33 – 0.69 | 0.43* | 0.18 | 0.08 – 0.79 | 0.14 – 0.73 | 0.46* | 0.18 | 0.11 – 0.82 | 0.16 – 0.75 |
| Envy | -0.55* | 0.11 | -0.76 – -0.32 | -0.73 – -0.36 | -0.73* | 0.18 | -1.08 – -0.39 | -1.02 – -0.44 | -0.75* | 0.19 | -1.12 – -0.39 | -1.06 – -0.45 |
| Self Interest | -0.38* | 0.11 | -0.60 – -0.17 | -0.56 – -0.20 | -0.53* | 0.18 | -0.88 – -0.19 | -0.83 – -0.24 | -0.56* | 0.18 | -0.93 – -0.21 | -0.87 – -0.27 |
| Age | 0.08* | 0.03 | 0.03 – 0.13 | 0.03 – 0.13 | 0.08* | 0.03 | 0.03 – 0.14 | 0.03 – 0.13 | 0.08* | 0.03 | 0.01 – 0.14 | 0.02 – 0.13 |
| Male | 0.03 | 0.09 | -0.15 – 0.20 | -0.12 – 0.18 | 0.04 | 0.09 | -0.15 – 0.21 | -0.11 – 0.19 | 0.04 | 0.10 | -0.15 – 0.22 | -0.12 – 0.20 |
| Magic*Envy |  |  |  |  | 0.51* | 0.26 | 0.01 – 1.00 | 0.08 – 0.92 | 0.55* | 0.26 | 0.03 – 1.07 | 0.12 – 0.98 |
| Religion*Envy |  |  |  |  | 0.06 | 0.25 | -0.44 – 0.56 | -0.35 – 0.48 | 0.04 | 0.25 | -0.48 – 0.55 | -0.38 – 0.46 |
| Magic*Self Int |  |  |  |  | 0.28 | 0.26 | -0.24 – 0.79 | -0.14 – 0.70 | 0.36 | 0.26 | -0.17 – 0.88 | -0.08 – 0.79 |
| Religion*Self Int |  |  |  |  | 0.19 | 0.26 | -0.32 – 0.67 | -0.23 – 0.60 | 0.18 | 0.25 | -0.34 – 0.68 | -0.24 – 0.59 |
| Education |  |  |  |  |  |  |  |  | 0.03 | 0.02 | -0.01 – 0.06 | 0.00 – 0.06 |
| Religiosity |  |  |  |  |  |  |  |  | 0.02 | 0.05 | -0.09 – 0.12 | -0.07 – 0.11 |
| Houses owned |  |  |  |  |  |  |  |  | 0.1 | 0.07 | -0.04 – 0.23 | -0.01 – 0.22 |
| Cars owned |  |  |  |  |  |  |  |  | -0.02 | 0.04 | -0.10 – 0.06 | -0.09 – 0.05 |
| English |  |  |  |  |  |  |  |  | 0.29 | 0.10 | 0.07 – 0.48 | 0.11 – 0.45 |
| Observations | 429 |  |  |  | 429 |  |  |  | 401 |  |  |  |

Table 3: Regression predicting perceived help or harm to community [-2 to +2 , 5 point scale] from vignette condition

| **Community** | **Model 1** | | | | **Model 2** | | | | **Model 3** | | | |
| --- | --- | --- | --- | --- | --- | --- | --- | --- | --- | --- | --- | --- |
| ***Predictors*** | ***Est.*** | ***SE*** | ***CI (95%)*** | ***CI (90%)*** | ***Est.*** | ***SE*** | ***CI (95%)*** | ***CI (90%)*** | ***Est.*** | ***SE*** | ***CI (95%)*** | ***CI (90%)*** |
| Intercept | 0.43* | 0.10 | 0.24 – 0.62 | 0.27 – 0.59 | 0.47* | 0.12 | 0.23 – 0.70 | 0.27 – 0.66 | 0.42* | 0.21 | 0.03 – 0.84 | 0.09 – 0.77 |
| Magic | -1.13* | 0.10 | -1.32 – -0.93 | -1.29 – -0.96 | -1.29* | 0.17 | -1.62 – -0.96 | -1.57 – -1.02 | -1.39* | 0.17 | -1.72 – -1.06 | -1.67 – -1.11 |
| Religion | 0.33* | 0.10 | 0.13 – 0.53 | 0.16 – 0.50 | 0.36* | 0.16 | 0.04 – 0.69 | 0.09 – 0.63 | 0.33† | 0.17 | -0.01 – 0.66 | 0.05 – 0.61 |
| Envy | -0.47* | 0.10 | -0.67 – -0.28 | -0.64 – -0.31 | -0.41* | 0.17 | -0.72 – -0.07 | -0.67 – -0.13 | -0.45* | 0.17 | -0.79 – -0.13 | -0.73 – -0.19 |
| Self Interest | -0.27* | 0.10 | -0.46 – -0.07 | -0.44 – -0.10 | -0.45* | 0.17 | -0.78 – -0.13 | -0.72 – -0.18 | -0.51* | 0.17 | -0.84 – -0.19 | -0.79 – -0.24 |
| Age | 0.04 | 0.03 | -0.02 – 0.09 | -0.01 – 0.08 | 0.04† | 0.02 | -0.01 – 0.09 | 0.00 – 0.08 | 0.04 | 0.03 | -0.02 – 0.09 | -0.01 – 0.09 |
| Male | 0.13 | 0.08 | -0.04 – 0.30 | -0.01 – 0.27 | 0.13 | 0.08 | -0.04 – 0.30 | -0.02 – 0.27 | 0.13 | 0.08 | -0.04 – 0.29 | -0.01 – 0.26 |
| Magic*Envy |  |  |  |  | 0.21 | 0.23 | -0.27 – 0.64 | -0.19 – 0.58 | 0.21 | 0.24 | -0.24 – 0.68 | -0.18 – 0.60 |
| Religion*Envy |  |  |  |  | -0.38 | 0.24 | -0.85 – 0.06 | -0.77 – -0.00 | -0.38 | 0.23 | -0.83 – 0.09 | -0.76 – 0.02 |
| Magic*Self Int |  |  |  |  | 0.30 | 0.24 | -0.16 – 0.76 | -0.08 – 0.68 | 0.40 | 0.23 | -0.06 – 0.86 | 0.01 – 0.79 |
| Religion*Self Int |  |  |  |  | 0.29 | 0.23 | -0.16 – 0.75 | -0.09 – 0.67 | 0.31 | 0.23 | -0.14 – 0.78 | -0.07 – 0.70 |
| Education |  |  |  |  |  |  |  |  | 0.01 | 0.02 | -0.02 – 0.04 | -0.01 – 0.04 |
| Religiosity |  |  |  |  |  |  |  |  | 0.05 | 0.05 | -0.05 – 0.14 | -0.03 – 0.13 |
| Houses owned |  |  |  |  |  |  |  |  | -0.09 | 0.06 | -0.21 – 0.03 | -0.19 – 0.01 |
| Cars owned |  |  |  |  |  |  |  |  | 0.02 | 0.04 | -0.05 – 0.09 | -0.04 – 0.08 |
| English |  |  |  |  |  |  |  |  | 0.11 | 0.09 | -0.07 – 0.30 | -0.04 – 0.26 |
| Observations | 429 |  |  |  | 429 |  |  |  | 401 |  |  |  |

### Trust and Character Rating Tables

Table 4: Correlation table for all character ratings

|  | 1 | 2 | 3 | 4 | 5 | 6 |
| --- | --- | --- | --- | --- | --- | --- |
| 1) Trust | 1 |  |  |  |  |  |
| 2) Helpful | 0.58 | 1 |  |  |  |  |
| 3) Harmful (R) | 0.56 | 0.46 | 1 |  |  |  |
| 4) Like | 0.75 | 0.50 | 0.48 | 1 |  |  |
| 5) Good | 0.71 | 0.55 | 0.55 | 0.73 | 1 |  |
| 6) Selfish (R) | 0.54 | 0.49 | 0.53 | 0.56 | 0.59 | 1 |

Table 5: Models for individual character ratings [-1.5 to 1.5, 4 point scale]

|  | **Helpful** | **Like** | **Good** | **(not) Harmful** | **(not) Selfish** |
| --- | --- | --- | --- | --- | --- |
| *Predictors* | *Est. (95% CI)* | *Est. (95% CI)* | *Est. (95% CI)* | *Est. (95% CI)* | *Est. (95% CI)* |
| Intercept | 0.36* | -0.09 | 0.06 | 0.52* | 0.31* |
|  | (0.18 – 0.54) | (-0.29 – 0.13) | (-0.15 – 0.28) | (0.33 – 0.69) | (0.10 – 0.53) |
| Magic | -0.85* | -0.71* | -0.61* | -0.85* | -0.69* |
|  | (-1.11 – -0.60) | (-1.00 – -0.42) | (-0.89 – -0.33) | (-1.09 – -0.60) | (-0.98 – -0.40) |
| Religion | -0.14 | 0.10 | 0.18 | 0.11 | 0.18 |
|  | (-0.38 – 0.11) | (-0.19 – 0.39) | (-0.10 – 0.47) | (-0.13 – 0.35) | (-0.13 – 0.47) |
| Envy | -0.42* | -0.34* | -0.19* | -0.21* | -0.37* |
|  | (-0.67 – -0.16) | (-0.63 – -0.05) | (-0.47 – 0.08) | (-0.46 – 0.04) | (-0.67 – -0.09) |
| Self Interest | -0.32* | -0.28 | -0.05 | 0.02 | -0.20 |
|  | (-0.56 – -0.06) | (-0.56 – 0.01) | (-0.33 – 0.22) | (-0.22 – 0.27) | (-0.50 – 0.08) |
| Age | 0.03 | 0.09* | 0.08* | -0.04* | 0.03 |
|  | (-0.00 – 0.07) | (0.04 – 0.13) | (0.04 – 0.12) | (-0.08 – -0.00) | (-0.01 – 0.08) |
| Male | 0.07 | 0.18* | 0.11 | 0.04 | 0.11 |
|  | (-0.05 – 0.19) | (0.04 – 0.32) | (-0.02 – 0.24) | (-0.08 – 0.17) | (-0.04 – 0.25) |
| Magic*Envy | 0.09 | 0.22 | -0.04 | 0.04 | 0.05 |
|  | (-0.27 – 0.44) | (-0.17 – 0.62) | (-0.42 – 0.35) | (-0.32 – 0.38) | (-0.36 – 0.47) |
| Religion*Envy | 0.15 | 0.04 | -0.27 | 0.11 | -0.37 |
|  | (-0.21 – 0.51) | (-0.36 – 0.45) | (-0.66 – 0.12) | (-0.26 – 0.46) | (-0.78 – 0.04) |
| Magic*Self Int. | 0.03 | 0.22 | -0.07 | -0.12 | 0.06 |
|  | (-0.32 – 0.39) | (-0.17 – 0.61) | (-0.45 – 0.30) | (-0.48 – 0.23) | (-0.34 – 0.46) |
| Religion*Self Int. | 0.13 | 0.14 | 0.06 | 0.18 | -0.13 |
|  | (-0.22 – 0.49) | (-0.25 – 0.55) | (-0.32 – 0.46) | (-0.16 – 0.54) | (-0.55 – 0.28) |
| Observations | 420 | 396 | 380 | 424 | 390 |

Table 6: Trust [-1.5 to 1.5, 4 point scale] predicted by condition

| **Trust** |  | | |  | |  | |  |  | |  | | |  | |  |  | | |  | |  | |  | |
| --- | --- | --- | --- | --- | --- | --- | --- | --- | --- | --- | --- | --- | --- | --- | --- | --- | --- | --- | --- | --- | --- | --- | --- | --- | --- |
| ***Predictors*** | ***Est.*** | | | ***SE*** | | ***CI (95%)*** | | ***CI (90%)*** | ***Est.*** | | ***SE*** | | | ***CI (95%)*** | | ***CI (90%)*** | ***Est.*** | | | ***SE*** | | ***CI (95%)*** | | ***CI (90%)*** | |
| Intercept | -0.08 | | | 0.08 | | -0.23 – 0.07 | | -0.21 – 0.05 | -0.01 | | 0.10 | | | -0.20 – 0.19 | | -0.17 – 0.15 | 0.21 | | | 0.17 | | -0.14 – 0.53 | | -0.08 – 0.48 | |
| Magic | -0.66* | | | 0.08 | | -0.82 – -0.50 | | -0.79 – -0.52 | -0.75* | | 0.14 | | | -1.02 – -0.48 | | -0.98 – -0.52 | -0.72* | | | 0.14 | | -1.00 – -0.44 | | -0.95 – -0.49 | |
| Religion | 0.14 | | | 0.08 | | -0.02 – 0.30 | | 0.01 – 0.27 | 0.03 | | 0.14 | | | -0.24 – 0.29 | | -0.19 – 0.25 | 0.01 | | | 0.15 | | -0.26 – 0.29 | | -0.21 – 0.25 | |
| Envy | -0.22* | | | 0.08 | | -0.37 – -0.07 | | -0.35 – -0.09 | -0.30* | | 0.13 | | | -0.57 – -0.04 | | -0.52 – -0.09 | -0.35* | | | 0.14 | | -0.63 – -0.08 | | -0.59 – -0.12 | |
| Self Interest | -0.08 | | | 0.08 | | -0.23 – 0.08 | | -0.21 – 0.05 | -0.21 | | 0.14 | | | -0.47 – 0.06 | | -0.43 – 0.01 | -0.24 | | | 0.14 | | -0.51 – 0.04 | | -0.47 – -0.01 | |
| Age | 0.06* | | | 0.02 | | 0.02 – 0.10 | | 0.03 – 0.09 | 0.06* | | 0.02 | | | 0.02 – 0.10 | | 0.03 – 0.09 | 0.06* | | | 0.02 | | 0.01 – 0.10 | | 0.02 – 0.10 | |
| Male | 0.07 | | | 0.07 | | -0.07 – 0.20 | | -0.04 – 0.17 | 0.07 | | 0.07 | | | -0.07 – 0.20 | | -0.04 – 0.18 | 0.07 | | | 0.07 | | -0.06 – 0.20 | | -0.04 – 0.18 | |
| Magic*Envy |  | | |  | |  | |  | 0.16 | | 0.19 | | | -0.22 – 0.53 | | -0.15 – 0.47 | 0.14 | | | 0.2 | | -0.25 – 0.53 | | -0.19 – 0.47 | |
| Religion*Envy |  | | |  | |  | |  | 0.08 | | 0.18 | | | -0.28 – 0.44 | | -0.22 – 0.38 | 0.13 | | | 0.2 | | -0.25 – 0.51 | | -0.20 – 0.45 | |
| Magic*Self Int |  | | |  | |  | |  | 0.12 | | 0.19 | | | -0.25 – 0.50 | | -0.19 – 0.44 | 0.09 | | | 0.2 | | -0.29 – 0.46 | | -0.24 – 0.40 | |
| Religion*Self Int | | |  | |  | |  | | | 0.27 | | 0.19 | -0.12 – 0.64 | | -0.06 – 0.58 | | | 0.30 | 0.19 | | -0.09 – 0.67 | | -0.03 – 0.61 | |  |
| Education | |  | |  | |  | |  |  | |  | | |  | |  | -0.02 | | | 0.01 | | -0.05 – 0.00 | | -0.04 – 0.00 | |
| Religiosity | |  | |  | |  | |  |  | |  | | |  | |  | 0.02 | | | 0.04 | | -0.05 – 0.10 | | -0.04 – 0.09 | |
| Houses owned | |  | |  | |  | |  |  | |  | | |  | |  | -0.02 | | | 0.05 | | -0.12 – 0.07 | | -0.10 – 0.06 | |
| Cars owned | |  | |  | |  | |  |  | |  | | |  | |  | 0.01 | | | 0.03 | | -0.05 – 0.07 | | -0.04 – 0.06 | |
| English | |  | |  | |  | |  |  | |  | | |  | |  | -0.11 | | | 0.08 | | -0.26 – 0.03 | | -0.23 – 0.01 | |
| Observations | | 413 | |  | |  | |  | 413 | |  | | |  | |  | 388 | | |  | |  | |  | |

Table 7: Multilevel model predicing character ratings [-1.5 to 1.5, 4 point scale] form condition with random intercepts for participants

| Character rating | Model 1 | | | | Model 2 | | | | Model 3 | | | |
| --- | --- | --- | --- | --- | --- | --- | --- | --- | --- | --- | --- | --- |
| ***Predictors*** | ***Est.*** | ***SE*** | ***CI (95%)*** | ***CI (90%)*** | ***Est.*** | ***SE*** | ***CI (95%)*** | ***CI (90%)*** | ***Est.*** | ***SE*** | ***CI (95%)*** | ***CI (90%)*** |
| Intercept | 0.23 | 0.13 | -0.08 – 0.60 | -0.01 – 0.50 | 0.25 | 0.13 | -0.04 – 0.58 | 0.02 – 0.51 | 0.23 | 0.15 | -0.13 – 0.59 | -0.04 – 0.51 |
| Magic | -0.72* | 0.04 | -0.79 – -0.64 | -0.77 – -0.65 | -0.77* | 0.07 | -0.90 – -0.64 | -0.87 – -0.66 | -0.77* | 0.07 | -0.90 – -0.64 | -0.88 – -0.66 |
| Religion | 0.09 | 0.04 | 0.01 – 0.16 | 0.03 – 0.15 | 0.07 | 0.06 | -0.06 – 0.20 | -0.04 – 0.17 | 0.06 | 0.07 | -0.07 – 0.19 | -0.05 – 0.17 |
| Envy | -0.30* | 0.04 | -0.38 – -0.23 | -0.37 – -0.24 | -0.33* | 0.07 | -0.45 – -0.20 | -0.43 – -0.22 | -0.39* | 0.07 | -0.52 – -0.25 | -0.50 – -0.28 |
| Self Interest | -0.13* | 0.04 | -0.21 – -0.06 | -0.19 – -0.07 | -0.18* | 0.07 | -0.31 – -0.05 | -0.29 – -0.08 | -0.22* | 0.07 | -0.35 – -0.10 | -0.34 – -0.12 |
| Age | 0.04* | 0.01 | 0.02 – 0.05 | 0.02 – 0.05 | 0.04* | 0.01 | 0.02 – 0.06 | 0.02 – 0.05 | 0.04* | 0.01 | 0.02 – 0.06 | 0.02 – 0.05 |
| Male | 0.10* | 0.03 | 0.04 – 0.16 | 0.05 – 0.15 | 0.10* | 0.03 | 0.04 – 0.16 | 0.05 – 0.15 | 0.09* | 0.03 | 0.03 – 0.15 | 0.03 – 0.14 |
| Magic*Envy |  |  |  |  | 0.10 | 0.09 | -0.08 – 0.27 | -0.05 – 0.25 | 0.14 | 0.09 | -0.04 – 0.32 | -0.01 – 0.29 |
| Religion*Envy |  |  |  |  | -0.05 | 0.09 | -0.22 – 0.14 | -0.19 – 0.11 | 0.02 | 0.09 | -0.16 – 0.20 | -0.13 – 0.17 |
| Magic*Self Int |  |  |  |  | 0.06 | 0.09 | -0.12 – 0.23 | -0.09 – 0.20 | 0.09 | 0.09 | -0.09 – 0.26 | -0.07 – 0.24 |
| Religion*Self Int |  |  |  |  | 0.10 | 0.09 | -0.08 – 0.28 | -0.05 – 0.26 | 0.13 | 0.09 | -0.05 – 0.31 | -0.02 – 0.28 |
| Education |  |  |  |  |  |  |  |  | 0.00 | 0.01 | -0.01 – 0.01 | -0.01 – 0.01 |
| Religiosity |  |  |  |  |  |  |  |  | 0.03† | 0.02 | -0.00 – 0.07 | 0.00 – 0.06 |
| Houses owned |  |  |  |  |  |  |  |  | 0.04 | 0.02 | -0.01 – 0.08 | -0.00 – 0.07 |
| Cars owned |  |  |  |  |  |  |  |  | 0.00 | 0.01 | -0.03 – 0.03 | -0.02 – 0.02 |
| English |  |  |  |  |  |  |  |  | 0.00 | 0.04 | -0.07 – 0.07 | -0.06 – 0.06 |
| **Random Effects** |  |  |  |  |  |  |  |  |  |  |  |  |
| σ^2^ | 0.46 |  |  |  | 0.46 |  |  |  | 0.46 |  |  |  |
| τ_00_ | 0.10 |  |  |  | 0.10 |  |  |  | 0.10 |  |  |  |
| ICC | 0.18 |  |  |  | 0.18 |  |  |  | 0.18 |  |  |  |
| N (Questions) | 5 |  |  |  | 5 |  |  |  | 5 |  |  |  |
| Observations | 2010 |  |  |  | 2010 |  |  |  | 1888 |  |  |  |

### Witchcraft accusation tables

Table 8: Logistic model (Bernoulli distribution) predicting suggestions of or claiming witchcraft by condition

| **Suggested** | **Model 1** | | | | **Model 2** | | | | **Model 3** | | | | |
| --- | --- | --- | --- | --- | --- | --- | --- | --- | --- | --- | --- | --- | --- |
| *Predictors* | ***OR*** | ***SE*** | ***CI (95%)*** | ***CI (90%)*** | ***OR*** | ***SE*** | ***CI (95%)*** | ***CI (90%)*** | ***OR*** | ***SE*** | ***CI (95%)*** | ***CI (90%)*** |  |
| Intercept | 0.21* | 0.07 | 0.11 – 0.39 | 0.12 – 0.35 | 0.22* | 0.07 | 0.11 – 0.40 | 0.13 – 0.37 | 0.20* | 0.14 | 0.05 – 0.84 | 0.06 – 0.69 |  |
| Magic | 16.65* | 5.32 | 8.91 – 32.98 | 9.87 – 29.65 | 14.19* | 5.60 | 6.32 – 32.02 | 7.28 – 27.85 | 13.09* | 5.58 | 5.62 – 30.86 | 6.38 – 26.67 |  |
| Religion | 0.53* | 0.17 | 0.29 – 0.99 | 0.32 – 0.90 | 0.55 | 0.23 | 0.22 – 1.32 | 0.26 – 1.16 | 0.52 | 0.23 | 0.21 – 1.25 | 0.24 – 1.07 |  |
| Envy | 3.17* | 1.07 | 1.67 – 6.06 | 1.88 – 5.53 | 2.66* | 1.00 | 1.27 – 5.76 | 1.42 – 5.06 | 2.59* | 1.03 | 1.17 – 5.82 | 1.32 – 5.08 |  |
| Self interest | 1.86† | 0.62 | 0.96 – 3.50 | 1.06 – 3.16 | 1.88 | 0.75 | 0.85 – 4.08 | 0.97 – 3.62 | 1.71 | 0.72 | 0.74 – 3.96 | 0.85 – 3.48 |  |
| Age | 0.89 | 0.08 | 0.75 – 1.05 | 0.77 – 1.03 | 0.88 | 0.08 | 0.75 – 1.04 | 0.77 – 1.02 | 0.83† | 0.08 | 0.68 – 1.01 | 0.70 – 0.98 |  |
| Male | 0.81 | 0.22 | 0.47 – 1.40 | 0.51 – 1.27 | 0.83 | 0.22 | 0.49 – 1.43 | 0.53 – 1.31 | 0.92 | 0.26 | 0.52 – 1.57 | 0.57 – 1.45 |  |
| Magic*Envy |  |  |  |  | 1.15 | 0.63 | 0.36 – 3.78 | 0.43 – 3.05 | 1.47 | 0.90 | 0.44 – 5.25 | 0.54 – 4.27 |  |
| Religion*Envy |  |  |  |  | 1.41 | 0.75 | 0.46 – 4.23 | 0.56 – 3.51 | 1.36 | 0.75 | 0.45 – 4.29 | 0.52 – 3.52 |  |
| Magic*Self int. |  |  |  |  | 1.59 | 0.89 | 0.52 – 5.29 | 0.63 – 4.38 | 1.81 | 1.06 | 0.58 – 6.17 | 0.69 – 5.02 |  |
| Religion*Self int. |  |  |  |  | 0.49 | 0.30 | 0.14 – 1.60 | 0.17 – 1.33 | 0.52 | 0.31 | 0.14 – 1.73 | 0.17 – 1.43 |  |
| Education |  |  |  |  |  |  |  |  | 0.96 | 0.05 | 0.87 – 1.07 | 0.88 – 1.05 |  |
| Religiosity |  |  |  |  |  |  |  |  | 1.03 | 0.16 | 0.75 – 1.42 | 0.79 – 1.35 |  |
| Houses owned |  |  |  |  |  |  |  |  | 1.16 | 0.31 | 0.68 – 1.92 | 0.75 – 1.78 |  |
| Cars owned |  |  |  |  |  |  |  |  | 1.01 | 0.15 | 0.76 – 1.31 | 0.79 – 1.27 |  |
| English |  |  |  |  |  |  |  |  | 0.95 | 0.30 | 0.51 – 1.82 | 0.56 – 1.62 |  |
| Observations | 341 |  |  |  | 341 |  |  |  | 319 |  |  |  |  |

Table 9: Logistic model (Bernoulli distribution) predicting direct claims of witchcraft by condition

| **Witchcraft** | **Model 1** | | | | **Model 2** | | | | **Model 3** | | | |
| --- | --- | --- | --- | --- | --- | --- | --- | --- | --- | --- | --- | --- |
| *Predictors* | ***OR*** | ***SE*** | ***CI (95%)*** | ***CI (90%)*** | ***OR*** | ***SE*** | ***CI (95%)*** | ***CI (90%)*** | ***OR*** | ***SE*** | ***CI (95%)*** | ***CI (90%)*** |
| Intercept | 0.15* | 0.05 | 0.08 – 0.30 | 0.09 – 0.27 | 0.14* | 0.05 | 0.07 – 0.29 | 0.08 – 0.26 | 0.09* | 0.07 | 0.02 – 0.40 | 0.02 – 0.32 |
| Magic | 5.59* | 1.78 | 2.95 – 11.14 | 3.29 – 9.82 | 6.18* | 2.55 | 2.67 – 14.26 | 3.04 – 12.59 | 5.82* | 2.47 | 2.42 – 14.14 | 2.85 – 12.21 |
| Religion | 0.36* | 0.16 | 0.14 – 0.86 | 0.16 – 0.74 | 0.40† | 0.20 | 0.14 – 1.06 | 0.17 – 0.90 | 0.36† | 0.19 | 0.12 – 1.01 | 0.14 – 0.87 |
| Envy | 1.08 | 0.36 | 0.57 – 2.11 | 0.63 – 1.91 | 1.33 | 0.57 | 0.55 – 3.13 | 0.64 – 2.75 | 1.28 | 0.59 | 0.51 – 3.25 | 0.59 – 2.75 |
| Self interest | 1.18 | 0.4 | 0.60 – 2.27 | 0.68 – 2.07 | 1.15 | 0.50 | 0.46 – 2.78 | 0.53 – 2.45 | 1.01 | 0.47 | 0.38 – 2.68 | 0.45 – 2.27 |
| Age | 0.78* | 0.08 | 0.64 – 0.95 | 0.66 – 0.92 | 0.78* | 0.08 | 0.64 – 0.93 | 0.66 – 0.91 | 0.74* | 0.08 | 0.59 – 0.91 | 0.61 – 0.88 |
| Male | 0.73 | 0.21 | 0.41 – 1.27 | 0.45 – 1.17 | 0.74 | 0.20 | 0.41 – 1.29 | 0.45 – 1.18 | 0.83 | 0.25 | 0.46 – 1.49 | 0.51 – 1.36 |
| Magic*Envy |  |  |  |  | 0.64 | 0.33 | 0.21 – 1.83 | 0.26 – 1.58 | 0.74 | 0.4 | 0.23 – 2.21 | 0.28 – 1.85 |
| Religion*Envy |  |  |  |  | 1.10 | 0.68 | 0.28 – 3.73 | 0.35 – 3.07 | 0.82 | 0.55 | 0.20 – 3.19 | 0.25 – 2.54 |
| Magic*Self int. |  |  |  |  | 1.18 | 0.63 | 0.41 – 3.62 | 0.48 – 2.96 | 1.38 | 0.74 | 0.45 – 4.12 | 0.55 – 3.48 |
| Religion*Self int. |  |  |  |  | 0.40 | 0.3 | 0.09 – 1.73 | 0.11 – 1.38 | 0.45 | 0.33 | 0.09 – 1.88 | 0.12 – 1.58 |
| Education |  |  |  |  |  |  |  |  | 0.96 | 0.06 | 0.85 – 1.06 | 0.87 – 1.05 |
| Religiosity |  |  |  |  |  |  |  |  | 1.22 | 0.21 | 0.89 – 1.71 | 0.93 – 1.62 |
| Houses owned |  |  |  |  |  |  |  |  | 1.44 | 0.4 | 0.82 – 2.40 | 0.89 – 2.24 |
| Cars owned |  |  |  |  |  |  |  |  | 1.11 | 0.17 | 0.82 – 1.48 | 0.86 – 1.42 |
| English |  |  |  |  |  |  |  |  | 0.92 | 0.31 | 0.47 – 1.77 | 0.53 – 1.58 |
| Observations | 341 |  |  |  | 341 |  |  |  | 319 |  |  |  |

## Study 2

## Vignettes

### Rahul – Normative

Rahul got a new job that paid very well. He only told close family and didn’t talk about his salary with anyone. He decided to buy a new car but waited a few months to buy it so he would not show off his increase in salary. About a week after he got his car, he got in a bad car accident and could not work anymore.

### Rahul – Envy

Rahul got a new job that paid very well. He told everyone about it and talked a lot about how much money he was going to make. When he got his first paycheque, he bought an expensive car. After about a week he got into a bad car accident and could not work anymore.

### Rahul – Selfish

Rahul got a new job that paid very well. He didn’t tell anyone about how much he was paid and decided to keep all the money for himself, even though some of his family members need money. He decided to buy a new car but waited a few months to buy it so he would not show off his increase in salary. About a week after he got his car, he got in a bad car accident and could not work anymore.

### Sandra – Normative

Sandra got a large sum of money from a family member who lived overseas. She only told close family and didn’t talk about the money with anyone. She bought only a couple of things that would not show off her new wealth. After a couple of months, she started to feel unwell. She was diagnosed with a chronic illness and was unable to enjoy all the things she had bought.

### Sandra – Envy

Sandra got a large sum of money from a family member who lived overseas. She told everyone about the money she got a bought a lot of expensive new clothing and other things. After a couple of months, she started to feel unwell. She was diagnosed with a chronic illness and was unable to enjoy all the expensive things she had bought.

### Sandra – Selfish

Sandra got a large sum of money from a family member who lived overseas. She didn’t tell anyone and even her family didn’t know. She decided to keep all the money for herself even though some of her other family members need money. She bought only a couple of things that would not show off her new wealth. After a couple of months, she started to feel unwell. She was diagnosed with a chronic illness and was unable to enjoy all the things she had bought.

### Ali – Normative

Ali got into a prestigious school overseas. He told only his close family about it and that he was likely to get a good job that paid well when he got home. A few weeks before he was supposed to leave, he had an accident and broke both of his legs. He was unable to leave and had to give up his place at the school.

### Ali – Envy

Ali got into a prestigious school overseas. He told everyone he met about it and the good job he would get and how much money he would make when he returned. A few weeks before he was supposed to leave he had an accident and broke both of his legs. He was unable to leave and had to give up his place at the school.

### Ali – Selfish

Ali got into a prestigious school overseas. He told only his close family, but secretly he planned to use his schooling to get a good job, but not use any of the money he made to help them. A few weeks before he was supposed to leave he had an accident and broke both of his legs. He was unable to leave and had to give up his place at the school.

## Questions

Questions were specific to each vignette. These are examples from the Rahul vignettes.

1. How likely is it that the car accident was Rahul’s fault? [Own Fault, 1-7, very unlikely to very likely]
2. How likely is it that the car accident was caused by God? [God, 1-7, very unlikely to very likely]
3. How likely is it that the car accident was caused by someone trying to harm Rahul through magic? [Magic, 1-7, very unlikely to very likely]
4. Do you think that Rahul telling people/not telling people about his job was good or bad? [Good/Bad, 1-7, very bad to very good]
5. If other people in Rahul’s community acted like Rahul did, how likely is it that they would come to harm? [Act Similar, 1-7, very unlikely to very likely]
6. How likely is it that would you trust members of Rahul’s community [Trust, 1-7 unlikely to very likely]

## Priors

$$\alpha\sim Normal\left( 0,1 \right)$$

$$\beta\sim Normal\left( 0,1 \right)$$

$$\sigma^{2}\sim Exponential(1)$$

## Tables

Table 10: How good or bad was the characters action predicted by condition

| **Good/Bad** |  |  |  |  |  |  |
| --- | --- | --- | --- | --- | --- | --- |
| *Predictors* | *Est.* | *CI (95%)* | *CI (90%)* | *Est.* | *CI (95%)* | *CI (90%)* |
| Intercept | 1.49* | 1.14 – 1.85 | 1.20 – 1.78 | 1.72* | 1.28 – 2.15 | 1.36 – 2.09 |
| Envy | -3.27* | -3.59 – -2.94 | -3.54 – -2.99 | -3.23* | -3.57 – -2.88 | -3.52 – -2.93 |
| Selfish | -0.97* | -1.29 – -0.64 | -1.24 – -0.69 | -0.94* | -1.29 – -0.60 | -1.23 – -0.66 |
| Age | -0.04 | -0.13 – 0.06 | -0.11 – 0.04 | 0.00 | -0.11 – 0.10 | -0.09 – 0.08 |
| Male | -0.02 | -0.33 – 0.29 | -0.28 – 0.24 | 0.01 | -0.30 – 0.32 | -0.24 – 0.26 |
| Ali | 0.22 | -0.11 – 0.55 | -0.07 – 0.50 | 0.25 | -0.11 – 0.60 | -0.06 – 0.54 |
| Sandra | 0.48* | 0.13 – 0.82 | 0.18 – 0.76 | 0.58* | 0.23 – 0.92 | 0.29 – 0.87 |
| Religiosity |  |  |  | -0.15* | -0.26 – -0.04 | -0.24 – -0.06 |
| Hindu |  |  |  | -0.37* | -0.72 – -0.00 | -0.67 – -0.07 |
| Muslim |  |  |  | 0.12 | -0.30 – 0.55 | -0.25 – 0.48 |
| **Random Effects** | |  |  |  |  |  |
| σ^2^ | 3.62 |  |  | 3.69 |  |  |
| τ_00_ | 0.18 |  |  | 0.12 |  |  |
| ICC | 0.05 |  |  | 0.03 |  |  |
| N | 244 |  |  | 224 |  |  |
| Observations | 724 |  |  | 666 |  |  |

Table 11: Was it the characters own fault predicted by condition

| **Own Fault** |  |  |  |  |  |  |
| --- | --- | --- | --- | --- | --- | --- |
| *Predictors* | *Est.* | *CI (95%)* | *CI (90%)* | *Est* | *CI (95%)* | *CI (90%)* |
| Intercept | -1.70* | -2.05 – -1.35 | -1.99 – -1.40 | -1.88* | -2.33 – -1.42 | -2.27 – -1.49 |
| Envy | 0.67* | 0.35 – 0.97 | 0.40 – 0.92 | 0.61* | 0.29 – 0.93 | 0.34 – 0.88 |
| Selfish | 0.91* | 0.60 – 1.22 | 0.64 – 1.18 | 0.84* | 0.51 – 1.17 | 0.57 – 1.12 |
| Age | 0.06 | -0.04 – 0.17 | -0.02 – 0.15 | 0.08 | -0.04 – 0.19 | -0.02 – 0.17 |
| Male | -0.03 | -0.35 – 0.31 | -0.30 – 0.26 | -0.04 | -0.38 – 0.31 | -0.33 – 0.26 |
| Ali | -0.46* | -0.77 – -0.15 | -0.72 – -0.20 | -0.43* | -0.76 – -0.10 | -0.70 – -0.15 |
| Sandra | -0.25 | -0.57 – 0.05 | -0.52 – -0.00 | -0.30 | -0.62 – 0.03 | -0.57 – -0.02 |
| Religiosity |  |  |  | 0.07 | -0.05 – 0.19 | -0.03 – 0.17 |
| Hindu |  |  |  | 0.23 | -0.17 – 0.63 | -0.10 – 0.57 |
| Muslim |  |  |  | 0.24 | -0.25 – 0.75 | -0.17 – 0.66 |
| **Random Effects** | |  |  |  |  |  |
| σ^2^ | 3.14 |  |  | 3.11 |  |  |
| τ_00_ | 0.69 |  |  | 0.68 |  |  |
| ICC | 0.18 |  |  | 0.18 |  |  |
| N | 244 |  |  | 224 |  |  |
| Observations | 722 |  |  | 666 |  |  |

Table 12: Would someone acting similar suffer a similar misfortune predicted by condition

| **Act Similar** |  |  |  |  |  |  |
| --- | --- | --- | --- | --- | --- | --- |
| *Predictors* | *Est.* | *CI (95%)* | *CI (90%)* | *Est.* | *CI (95%)* | *CI (90%)* |
| Intercept | -1.43* | -1.79 – -1.07 | -1.74 – -1.13 | -1.51* | -2.01 – -1.00 | -1.94 – -1.08 |
| Envy | 0.73* | 0.46 – 1.02 | 0.50 – 0.98 | 0.73* | 0.44 – 1.04 | 0.49 – 0.99 |
| Selfish | 0.47* | 0.18 – 0.76 | 0.23 – 0.72 | 0.52* | 0.22 – 0.81 | 0.26 – 0.77 |
| Age | 0.08 | -0.04 – 0.21 | -0.02 – 0.19 | 0.11 | -0.03 – 0.25 | -0.01 – 0.23 |
| Male | -0.04 | -0.43 – 0.35 | -0.37 – 0.28 | 0.05 | -0.35 – 0.47 | -0.28 – 0.40 |
| Ali | 0.07 | -0.23 – 0.35 | -0.18 – 0.30 | 0.08 | -0.22 – 0.40 | -0.17 – 0.35 |
| Sandra | 0.00 | -0.28 – 0.28 | -0.23 – 0.23 | -0.04 | -0.34 – 0.26 | -0.28 – 0.21 |
| Religiosity |  |  |  | 0.18* | 0.03 – 0.32 | 0.06 – 0.30 |
| Hindu |  |  |  | -0.29 | -0.79 – 0.20 | -0.69 – 0.13 |
| Muslim |  |  |  | -0.01 | -0.58 – 0.57 | -0.49 – 0.47 |
| **Random Effects** | |  |  |  |  |  |
| σ^2^ | 2.64 |  |  | 2.73 |  |  |
| τ_00_ | 1.74 |  |  | 1.63 |  |  |
| ICC | 0.40 |  |  | 0.37 |  |  |
| N | 244 |  |  | 224 |  |  |
| Observations | 726 |  |  | 669 |  |  |

Table 13: Did God cause the misfortune predicted by condition

| **God** |  |  | |  | |  | |  | |  | |  |
| --- | --- | --- | --- | --- | --- | --- | --- | --- | --- | --- | --- | --- |
| *Predictors* | *Est.* | *CI (95%)* | | *CI (90%)* | | *Est.* | | *CI (95%)* | | *CI (90%)* | |  |
| Intercept | -1.65* | -2.02 – -1.27 | | -1.96 – -1.32 | | -2.47* | | -2.97 – -1.98 | | -2.88 – -2.06 | |  |
| Envy | 0.10 | -0.14 – 0.33 | | -0.10 – 0.29 | | 0.10 | | -0.14 – 0.34 | | -0.11 – 0.30 | |  |
| Selfish | 0.26* | 0.02 – 0.49 | | 0.06 – 0.45 | | 0.29* | | 0.04 – 0.54 | | 0.08 – 0.50 | |  |
| Age | -0.12† | -0.27 – 0.02 | | -0.24 – -0.00 | | -0.13† | | -0.27 – 0.01 | | -0.24 – -0.01 | |  |
| Male | 0.11 | -0.34 – 0.56 | | -0.28 – 0.49 | | -0.03 | | -0.44 – 0.41 | | -0.36 – 0.33 | |  |
| Ali | 0.26* | 0.03 – 0.50 | | 0.06 – 0.46 | | 0.27* | | 0.02 – 0.50 | | 0.06 – 0.47 | |  |
| Sandra | 0.19 | -0.04 – 0.42 | | -0.01 – 0.38 | | 0.22† | | -0.01 – 0.46 | | 0.03 – 0.43 | |  |
| Religiosity |  |  | |  | | 0.15* | | 0.00 – 0.29 | | 0.02 – 0.26 | |  |
| Hindu |  |  | |  | | 0.40† | | -0.06 – 0.88 | | 0.01 – 0.80 | |  |
| Muslim |  |  | |  | | 2.36* | | 1.79 – 2.90 | | 1.88 – 2.83 | |  |
| **Random Effects** | | |  | |  | |  | |  | |  | |
| σ^2^ | 1.73 |  | |  | | 1.74 | |  | |  | |  |
| τ_00_ | 2.94 |  | |  | | 2.00 | |  | |  | |  |
| ICC | 0.63 |  | |  | | 0.53 | |  | |  | |  |
| N | 244 |  | |  | | 224 | |  | |  | |  |
| Observations | 722 |  | |  | | 664 | |  | |  | |  |

Table 14: Did someone doing magic cause the misfortune predicted by condition

| **Magic** |  |  | |  | |  | |  | | |  | | |  |  |
| --- | --- | --- | --- | --- | --- | --- | --- | --- | --- | --- | --- | --- | --- | --- | --- |
| *Predictors* | *Est.* | *CI (95%)* | | *CI (90%)* | | *Est.* | | *CI (95%)* | | | *CI (90%)* | | |  |  |
| Intercept | -1.76* | -2.07 – -1.44 | | -2.02 – -1.49 | | -1.65* | | -2.09 – -1.19 | | | -2.01 – -1.26 | | |  |  |
| Envy | 0.44* | 0.24 – 0.64 | | 0.27 – 0.61 | | 0.42* | | 0.21 – 0.63 | | | 0.24 – 0.60 | | |  |  |
| Selfish | -0.15 | -0.36 – 0.05 | | -0.32 – 0.02 | | -0.17 | | -0.39 – 0.04 | | | -0.35 – 0.01 | | |  |  |
| Age | -0.10† | -0.22 – 0.01 | | -0.20 – -0.01 | | -0.15* | | -0.27 – -0.02 | | | -0.25 – -0.04 | | |  |  |
| Male | -0.19 | -0.56 – 0.20 | | -0.50 – 0.14 | | -0.19 | | -0.56 – 0.20 | | | -0.49 – 0.15 | | |  |  |
| Ali | -0.10 | -0.30 – 0.10 | | -0.26 – 0.07 | | -0.11 | | -0.32 – 0.10 | | | -0.28 – 0.07 | | |  |  |
| Sandra | -0.14 | -0.34 – 0.06 | | -0.31 – 0.03 | | -0.21 | | -0.41 – 0.01 | | | -0.38 – -0.03 | | |  |  |
| Religiosity |  |  | |  | | 0.12† | | -0.01 – 0.24 | | | 0.01 – 0.22 | | |  |  |
| Hindu |  |  | |  | | -0.35 | | -0.81 – 0.10 | | | -0.72 – 0.02 | | |  |  |
| Muslim |  |  | |  | | -0.33 | | -0.86 – 0.20 | | | -0.78 – 0.12 | | |  |  |
| **Random Effects** | | |  | |  | |  | | |  | | |  | | |
| σ^2^ | 1.3 |  | |  | | 1.26 | | |  | | |  | | |  |
| τ_00_ | 1.89 |  | |  | | 1.77 | | |  | | |  | | |  |
| ICC | 0.59 |  | |  | | 0.58 | | |  | | |  | | |  |
| N | 244 |  | |  | | 224 | | |  | | |  | | |  |
| Observations | 726 |  | |  | | 668 | | |  | | |  | | |  |

Table 15: How much would you trust someone in the community predicted by condition

| **Trust** |  |  |  |  |  |  |
| --- | --- | --- | --- | --- | --- | --- |
| *Predictors* | *Est.* | *CI (95%)* | *CI (90%)* | *Est.* | *CI (95%)* | *CI (90%)* |
| Intercept | -1.63* | -1.96 – -1.32 | -1.90 – -1.37 | -1.82* | -2.28 – -1.36 | -2.20 – -1.43 |
| Envy | -0.10 | -0.28 – 0.07 | -0.25 – 0.05 | -0.07 | -0.26 – 0.12 | -0.23 – 0.09 |
| Selfish | -0.10 | -0.28 – 0.07 | -0.25 – 0.04 | -0.06 | -0.26 – 0.13 | -0.22 – 0.10 |
| Age | 0.04 | -0.07 – 0.17 | -0.05 – 0.15 | 0.07 | -0.06 – 0.20 | -0.04 – 0.17 |
| Male | 0.64* | 0.25 – 1.01 | 0.31 – 0.95 | 0.62* | 0.23 – 1.04 | 0.29 – 0.97 |
| Ali | 0.08 | -0.09 – 0.27 | -0.06 – 0.24 | 0.12 | -0.06 – 0.31 | -0.03 – 0.28 |
| Sandra | 0.12 | -0.06 – 0.30 | -0.03 – 0.27 | 0.17 | -0.01 – 0.36 | 0.02 – 0.33 |
| Religiosity |  |  |  | -0.02 | -0.16 – 0.12 | -0.13 – 0.10 |
| Hindu |  |  |  | 0.38* | -0.07 – 0.85 | 0.00 – 0.77 |
| Muslim | |  |  | 0.06 | -0.49 – 0.61 | -0.40 – 0.53 |
| **Random Effects** | |  |  |  |  |  |
| σ^2^ | 0.97 |  |  | 1.01 |  |  |
| τ_00_ | 2.14 |  |  | 2.17 |  |  |
| ICC | 0.69 |  |  | 0.68 |  |  |
| N | 244 |  |  | 224 |  |  |
| Observations | 723 |  |  | 667 |  |  |
